# Supplementary material for: Xbp1s-FoxO1 axis governs lipid accumulation and contractile performance in heart failure with preserved ejection fraction
Source: Nat Commun. 2021 Mar 16;12:1684. doi: 10.1038/s41467-021-21931-9 (PMC7966396; doi:10.1038/s41467-021-21931-9)

SUPPLEMENTARY INFORMATION

Xbp1s-FoxO1 Axis Governs Lipid Accumulation and Contractile Performance in Heart Failure with Preserved Ejection Fraction

Gabriele G. Schiattarella[1,2,3,4,5], Francisco Altamirano[1], Soo Young Kim[1], Dan Tong[1], Anwarul Ferdous[1], Hande Piristine[1], Subhajit Dasgupta[1], Xuliang Wang[1], Kristin M. French[1], Elisa Villalobos[1], Stephen B. Spurgin[6], Maayan Hotiner Waldman[1], Nan Jiang[1], Herman I. May[1], Theodore M. Hill[1], Yuxuan Luo[1], Heesoo Yoo[1], Vlad Zaha[1], Sergio Lavandero[1,7], Thomas G. Gillette[1], Joseph A. Hill[1,8]

[1]Department of Internal Medicine (Cardiology), University of Texas Southwestern Medical Center, Dallas, Texas, USA

[2]Department of Advanced Biomedical Sciences, Federico II University, Naples, Italy

[3]Center for Cardiovascular Research (CCR), Department of Cardiology, Charité - Universitätsmedizin Berlin, Berlin, Germany

[4]DZHK (German Centre for Cardiovascular Research), Partner Site Berlin, Berlin, Germany

[5]Translational Approaches in Heart Failure and Cardiometabolic Disease, Max Delbrück Center for Molecular Medicine in the Helmholtz Association (MDC), Berlin, Germany

[6]Department of Pediatrics, University of Texas Southwestern Medical Center, Dallas, Texas, USA,

[7]Advanced Center for Chronic Diseases (ACCDiS) & Center for Molecular Studies of the Cell (CEMC), Faculty of Chemical & Pharmaceutical Sciences & Faculty of Medicine, University of Chile, Santiago, Chile

[8]Department of Molecular Biology, University of Texas Southwestern Medical Center, Dallas, Texas, USA,

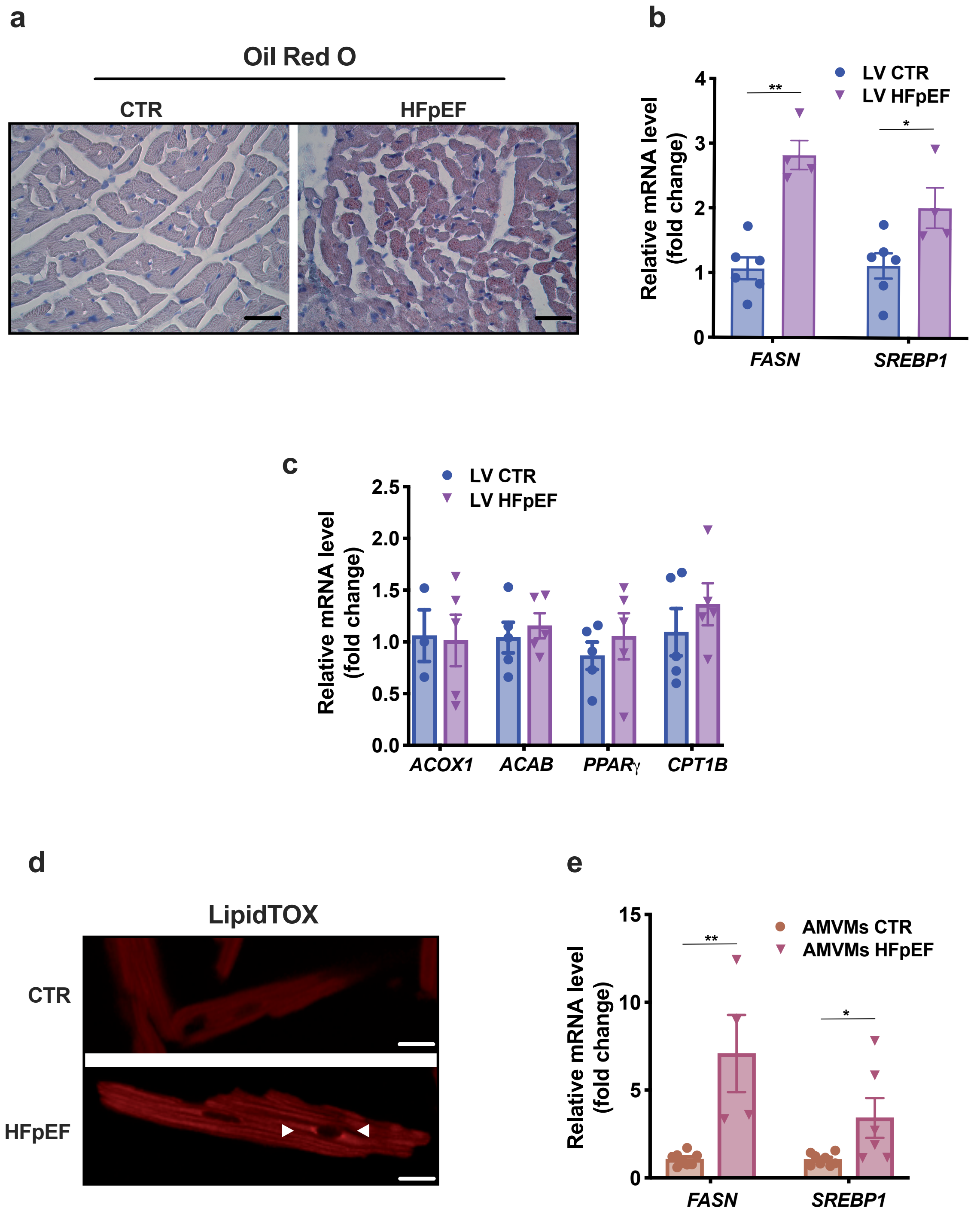

Supplementary Figure 1 | See next page for caption

Supplementary Figure 1 │ Cardiac lipid accumulation in hearts and cardiomyocytes from HFpEF mice. a, Representative images of Oil Red O staining of left ventricular (LV) sections from control (CTR) and HFpEF hearts. Scale bars = 50 μm. Images are representative of 4 hearts/group. **b,** mRNA levels of *FASN* and *SREBP1* in LV of CTR and HFpEF mice (n=6 for CTR group and n=4 for HFpEF group). **c,** mRNA levels of *ACOX1*, *ACAB*, *PPAR*γ, *CPT1B* – genes involved in lipid oxidation – in LV of CTR and HFpEF mice (n=3 for *ACOX* CTR group and n=5 for the remaining groups). **d,** LipidTox™ dye staining of isolated adult mouse ventricular myocytes (AMVMs) of CTR and HFpEF mice. Arrowheads indicate perinuclear lipid accumulation. Scale bars = 10 μm. Images are representative of 4 AMVMs preparations/group. **e,** mRNA levels of *FASN* and *SREBP1* in AMVMs of CTR and HFpEF mice (n=8 for CTR group; n=4 for *FASN* HFpEF group and n=6 for *SREBP1* HFpEF group). Results are presented as mean ± S.E.M. In b and e *p<0.05, **p<0.005, unpaired, two-tailed Kolmogorov-Smirnov test.

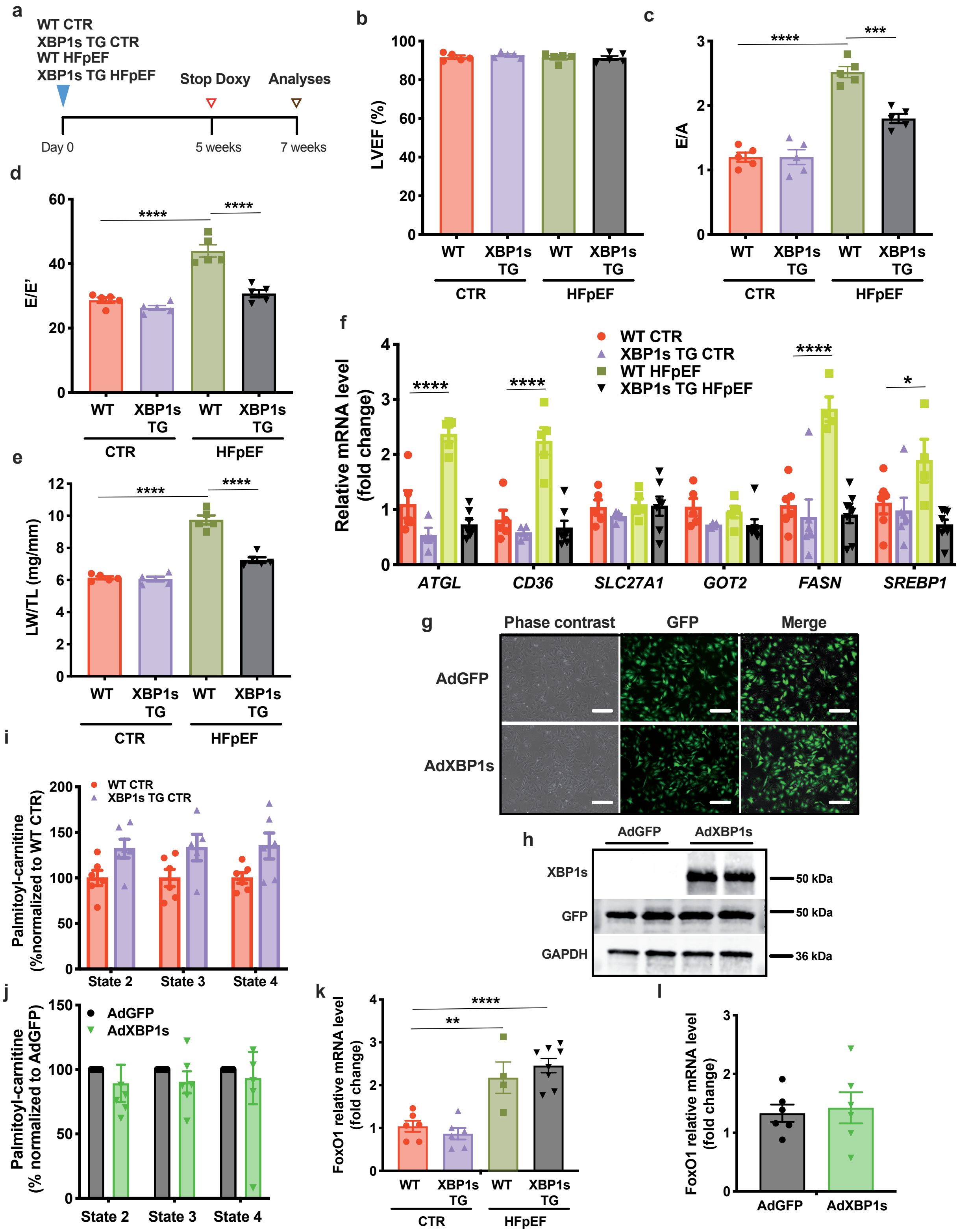

Supplementary Figure 2 | See next page for caption

Supplementary Figure 2 │ Cardiomyocyte-specific Xbp1s transgenic mice display ameliorated HFpEF phenotype, and Xbp1s over-expression in cardiomyocytes does not affect mitochondrial lipid utilization. a, Experimental design. WT and Xbp1s TG mice TG were exposed to CTR or HFpEF diet (blue triangle). After five weeks, doxycycline (Doxy) was removed from the drinking water to induce transgene expression (red triangle). Two weeks after transgene induction (brown triangle), mice were subjected to functional analysis and tissue harvesting. **b,** LVEF% of different experimental groups (n=5/group). **c,** E/A ratio of different experimental cohorts (n=5/group). **d**, E/E' ratio of different experimental cohorts (n=5/group). **e**, Ratio between wet lung weight (LW) and tibia length (TL) of different experimental cohorts (n=5/group). **f,** mRNA levels of *ATGL*, *CD36*, *SLC27A1*, *GOT2*, *FASN*, *SREBP1* in LV of WT and Xbp1s TG CTR and HFpEF hearts (n=5 for *ATGL*, CD36, *SLC27A1*, *GOT2* WT CTR group and WT HFpEF group, n=6 for *FASN*, *SREBP1* WT CTR group and Xbp1s TG CTR group, n=4 for *FASN*, *SREBP1* WT HFpEF group, n=7 for *ATGL*, *CD36*, *SLC27A1*, *GOT2* Xbp1s TG HFpEF group and n=8 for *FASN*, *SREBP1* Xbp1s TG HFpEF group). **g,** Representative images of phase contrast, GFP and merge of NRVMs transduced with AdGFP and AdXbp1s. Scale bars = 50 μm. Images are representative of 4 biologically independent experiments. **h,** Representative immunoblot images of Xbp1s, GFP and GAPDH proteins from NRVMs transduced with AdGFP or AdXbp1s. Images are representative of 4 biologically independent experiments. **i,** Oxygen consumption rates of isolated mitochondria from WT and Xbp1s TG CTR hearts. Mitochondria were exposed to palmitoylcarnitine as substrate. State 2 is the basal level, state 3 is the maximal level after adding ADP, and state 4 is the level after ADP exhaustion (n=5 for State 3 Xbp1s TG group and n=6 for for the remaining groups). **j,** Oxygen consumption rates of isolated mitochondria from NRVMs transduced with AdGFP or AdXbp1s (n=6 biologically independent experiments). **k,** mRNA level of *FoxO1* in LV of WT and Xbp1s TG CTR and HFpEF hearts (n=6 for WT CTR group and Xbp1s TG CTR group, n=4 for WT HFpEF group and n=8 for Xbp1s TG HFpEF group). **l,** mRNA levels of *FoxO1* in NRVMs transduced with AdGFP or AdXbp1s (n=6 biologically independent experiments). Results are presented as mean ± S.E.M. In c, d, e, k **p<0.005, ***p<0.0005, ****p<0.0001, 2-way ANOVA plus Sidak's multiple comparisons test. In f *p=0.015, ****p<0.0001, 1-way ANOVA plus Sidak's multiple comparisons test.

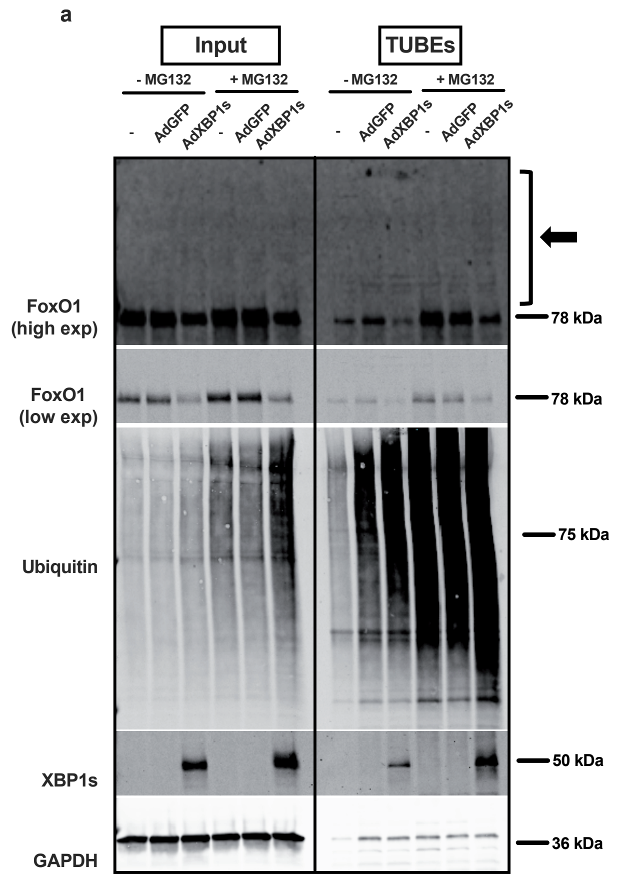

Supplementary Figure 3 │ Xbp1s-dependent ubiquitination of FoxO1 in cardiomyocytes. a, Representative immunoblot images of FoxO1, Ubiquitin, Xbp1s and GAPDH proteins from NRVMs transduced with AdGFP, AdXbp1s, or not transduced (-) in the presence or absence of MG132. Tandem Ubiquitin Binding Entities (TUBEs) magnetic beads were used to specifically detect polyubiquitin moieties. Arrow indicates FoxO1 polyubiquitination protein smear. Images are representative of 4 biologically independent experiments.

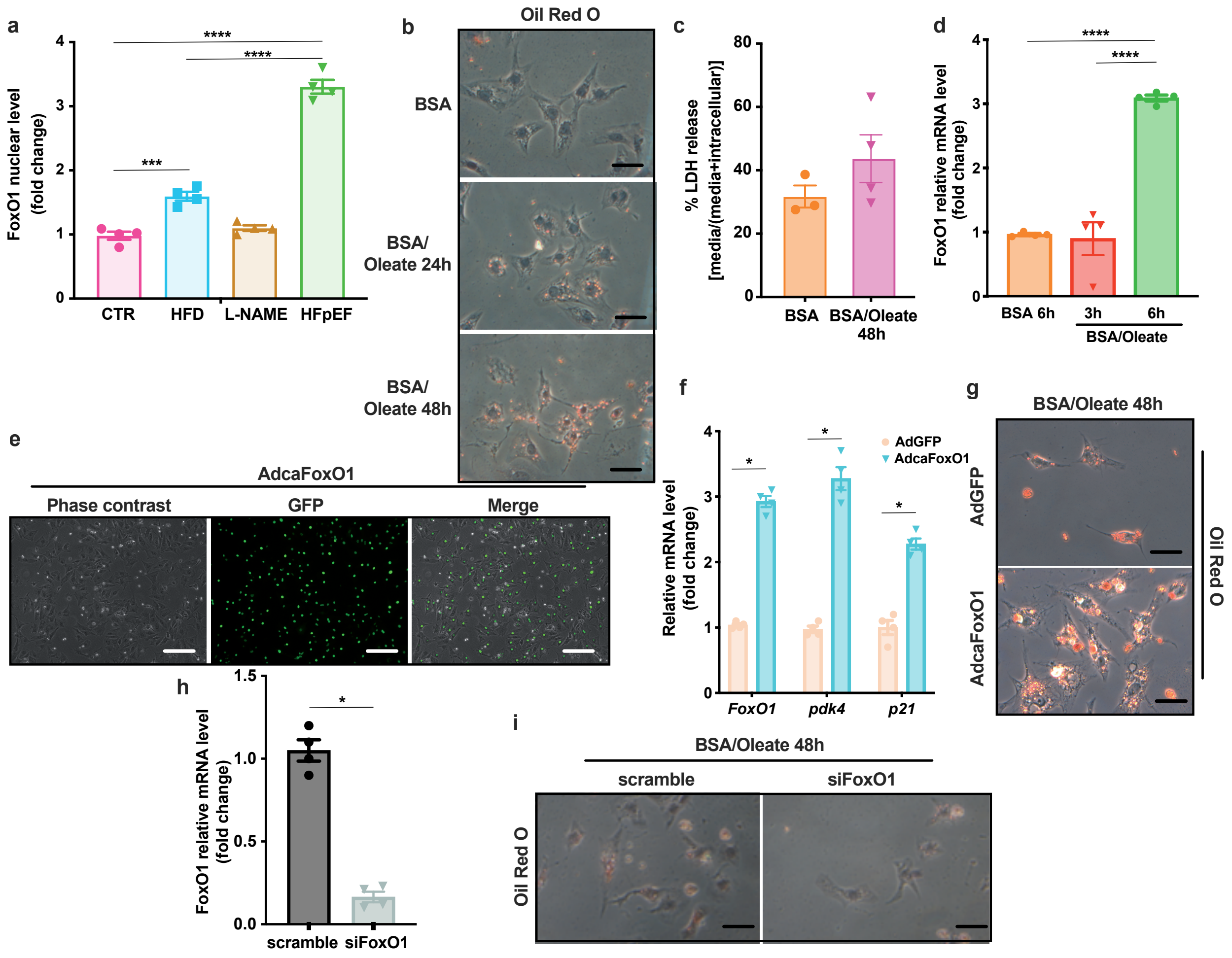

Supplementary Figure 4 | See next page for caption

Supplementary Figure 4 │ Feed-forward control of lipids by FoxO1 in cardiomyocytes. a, Bar graphs depicting nuclear FoxO1 protein levels in the different experimental groups of mice (n=4/group). **b,** Representative images of Oil Red O staining of NRVMs treated for 24h or 48h with either bovine serum albumin (BSA) or BSA/Oleate complex. Scale bars = 5 μm. Images are representative of 6 biologically independent experiments. **c,** Lactate dehydrogenase (LDH) release in NRVMs treated with BSA or BSA/Oleate complex for 48h (n=4 biologically independent experiments). **d,** mRNA level of *FoxO1* in NRVMs treated with BSA or BSA/Oleate complex for 3h and 6h (n=4 biologically independent experiments). **e,** Representative images of phase contrast, GFP and merge of NRVMs transduced with adenovirus expressing constitutively active FoxO1 (AdcaFoxO1). Scale bars = 50 μm. Images are representative of 4 biologically independent experiments **f,** mRNA levels of *FoxO1*, *pdk4* and *p21* in NRVMs transduced with AdGFP or AdcaFoxO1 (n=4 biologically independent experiments). **g,** Representative images of Oil Red O staining of NRVMs transduced with AdGFP or AdcaFoxO1 and treated for 48h with BSA/Oleate complex. Scale bars = 5 μm. Images are representative of 4 biologically independent experiments. **h,** mRNA level of *FoxO1* in NRVMs transfected siFoxO1 or scrambled siRNA control (n=4 biologically independent experiments). **i,** Representative images of Oil Red O staining of NRVMs transfected with siFoxO1 or scrambled siRNA and treated for 48h with BSA/Oleate complex. Scale bars = 5 μm. Images are representative of 3 biologically independent experiments. Results are presented as mean ± S.E.M. In a,d, ***$p<0.0005$, ****$p<0.0001$, 1-way ANOVA plus Sidak's multiple comparisons test. In f, h *$p<0.05$, unpaired, two-tailed Kolmogorov-Smirnov test.

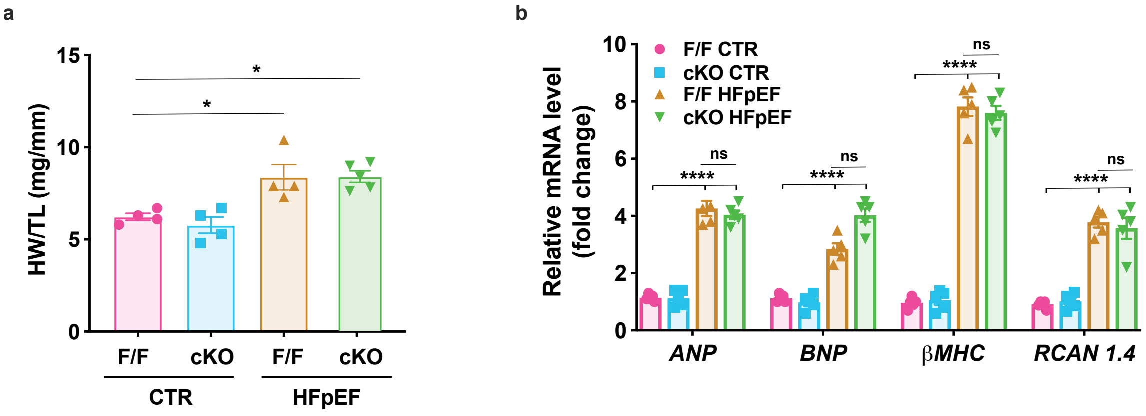

Supplementary Figure 5 │ Similar degree of cardiac hypertrophy in cardiomyocyte-specific FoxO1 knockout mice and wild-type mice under HFpEF conditions. a, Heart weight to tibia length ratio (HW/TL) in FoxO1 F/F and FoxO1 cKO CTR and HFpEF mice (n=4 for F/F CTR, cKO CTR and F/F HFpEF groups; n=5 for cKO HFpEF group). **b,** mRNA levels of *ANP*, *BNP*, *βMHC*, *RCAN1.4* in LV of FoxO1 F/F and FoxO1 cKO CTR and HFpEF hearts (n=5/group). Results are presented as mean ± S.E.M. In a, b *p<0.05, ****p<0.0001, 2-way ANOVA plus Sidak's multiple comparisons test.

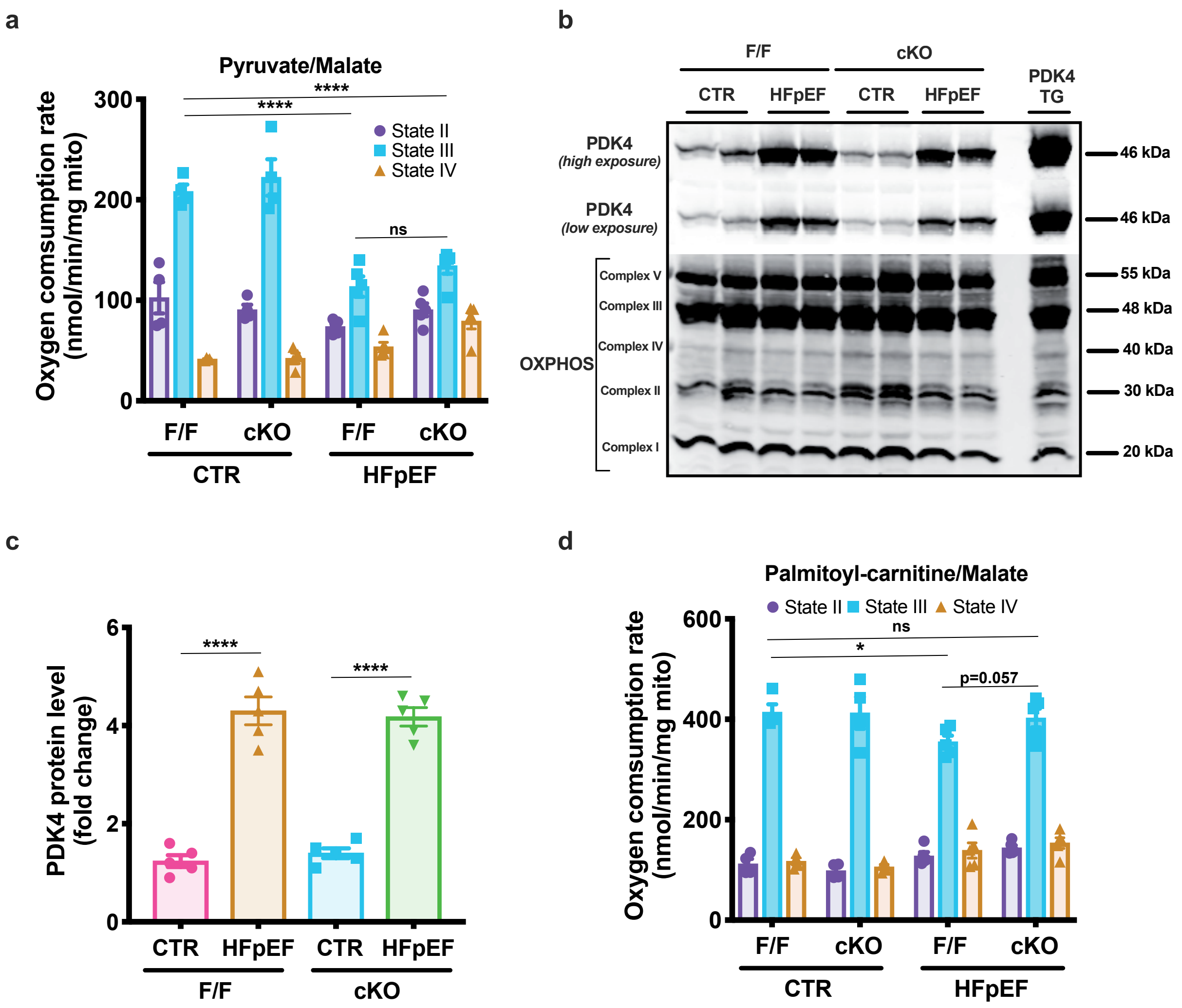

Supplementary Figure 6 | See next page for caption

Supplementary Figure 6 │ Mitochondrial oxidation in FoxO1 F/F and cKO hearts subjected to HFpEF. a, Maximal pyruvate-dependent oxygen consumption rates of isolated mitochondria from FoxO1 F/F and FoxO1 cKO hearts under CTR and HFpEF conditions (n=4 for F/F CTR, F/F HFpEF and cKO CTR groups; n=5 for cKO HFpEF group). **b,** Representative immunoblot images of PDK4 and OXPHPS proteins from FoxO1 F/F and FoxO1 cKO hearts under CTR and HFpEF conditions. Images are representative of 5 hearts/group. **c,** Densitometric analysis of PDK4 protein band in the different experimental groups (n=5/group). **d,** Maximal palmitoylcarnitine-dependent oxygen consumption rates of isolated mitochondria from FoxO1 F/F and FoxO1 cKO hearts under CTR and HFpEF conditions (n=4 for F/F CTR, F/F HFpEF and cKO CTR groups; n=5 for cKO HFpEF group). Results are presented as mean ± S.E.M. In a, c, d *p=0.024, ****p<0.0001, 2-way ANOVA plus Sidak's multiple comparisons test.

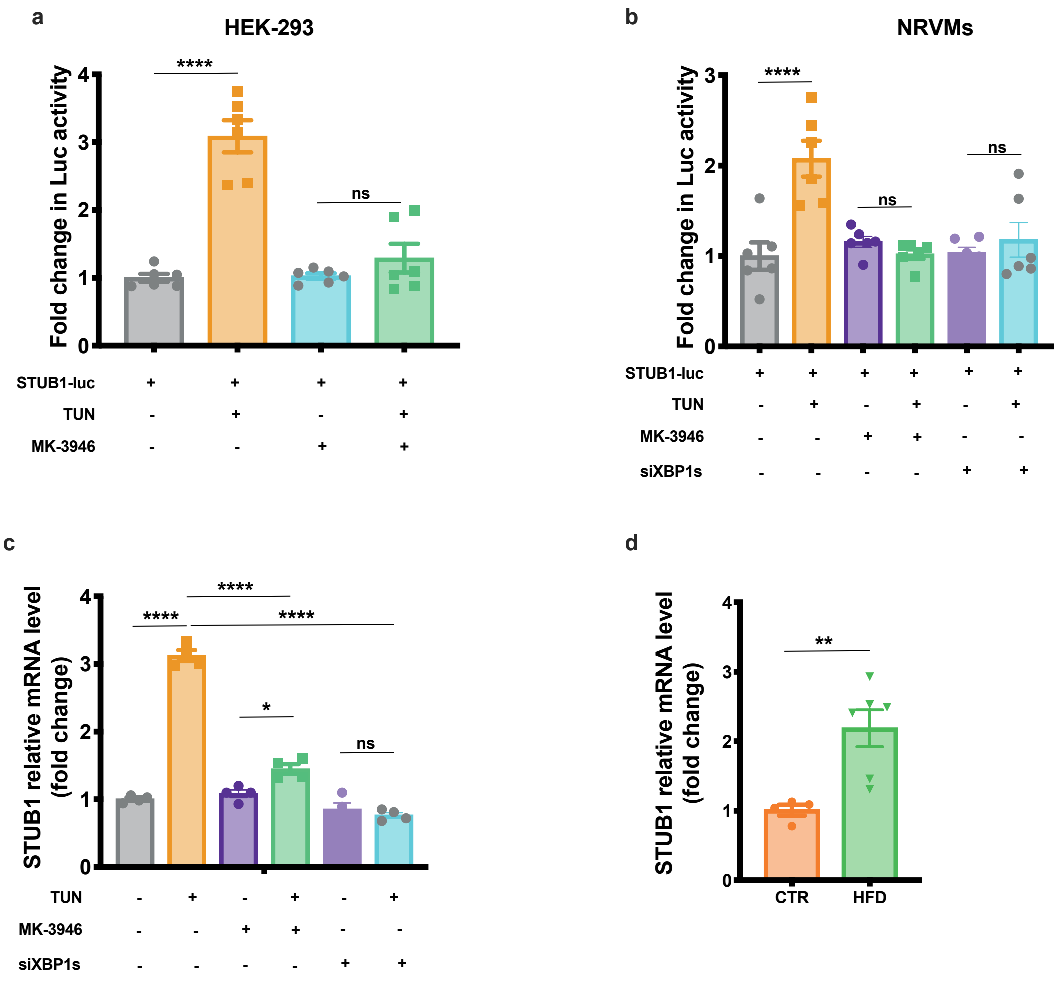

Supplementary Figure 7 | See next page for caption

Supplementary Figure 7 │ STUB1 levels are increased upon tunicamycin treatment in a Xbp1s-dependent manner. a, Luciferase (Luc) activity in HEK 293 transfected with STUB1 luciferase reporter construct (STUB1-Luc) and treated with tunicamycin (TUN) and MK-3946 (n=6 biologically independent experiments). **b,** Luc activity in NRVMs transfected with STUB1-Luc and treated with TUN, MK-3946 and siXbp1s (n=6 biologically independent experiments). **c,** mRNA levels of *STUB1* in NRVMs treated with TUN, MK-3946 and siXbp1s (n=4 biologically independent experiments). **d,** mRNA level of *STUB1*, in LV of CTR and HFD mice (n=4 for CTR group and n=6 for HFD group). Results are presented as mean ± S.E.M. In a, b, c *p=0.0147, ****p<0.0001, 2-way ANOVA plus Sidak's multiple comparisons test. In d **p<0.005, unpaired, two-tailed Kolmogorov-Smirnov test.

a

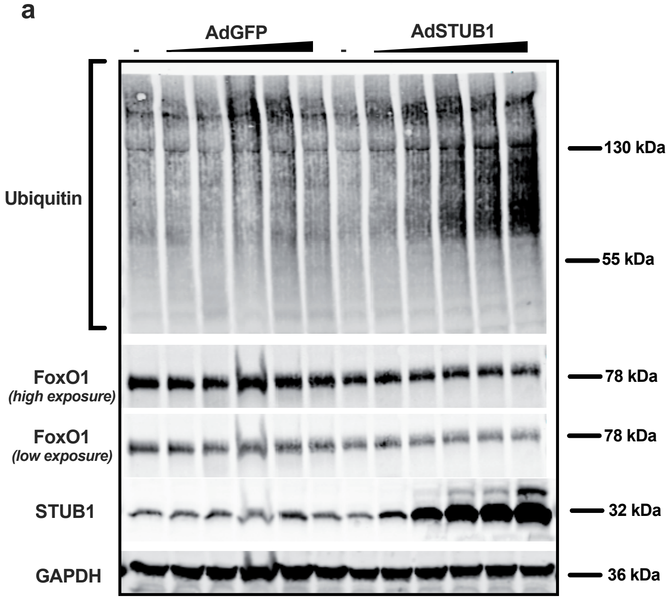

b

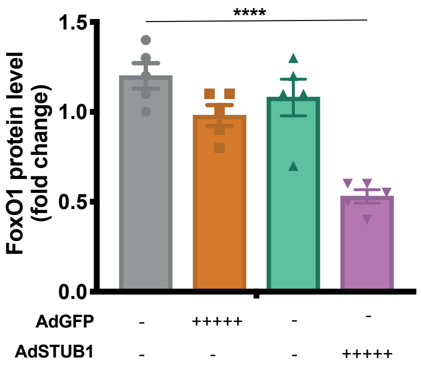

c

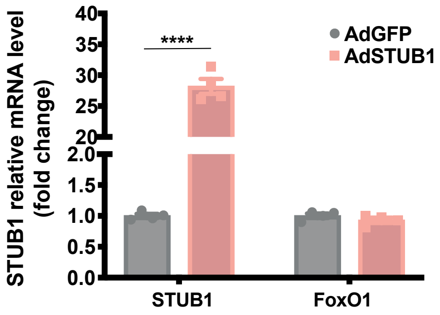

Supplementary Figure 8 | See next page for caption

Supplementary Figure 8 │ STUB1-dependent FoxO1 degradation in cardiomyocytes. a, Representative immunoblot images of Ubiquitin, FoxO1, STUB1 and GAPDH proteins from NRVMs transduced with increasing multiplicity of infection of AdGFP, adenovirus encoding for STUB1 (AdSTUB1) or not transduced (-). Images are representative of 5 independent experiments **b,** Densitometric analysis of FoxO1 protein band in the different experimental groups (n=5 biologically independent experiments). **c,** mRNA levels of *STUB1* and *FoxO1* in NRVMs transduced with AdGFP or AdSTUB1 (n=4 biologically independent experiments). Results are presented as mean ± S.E.M. In b ****p<0.0001, 1-way ANOVA plus Sidak's multiple comparisons test. In c ****p<0.0001, unpaired, two-tailed Kolmogorov-Smirnov test.

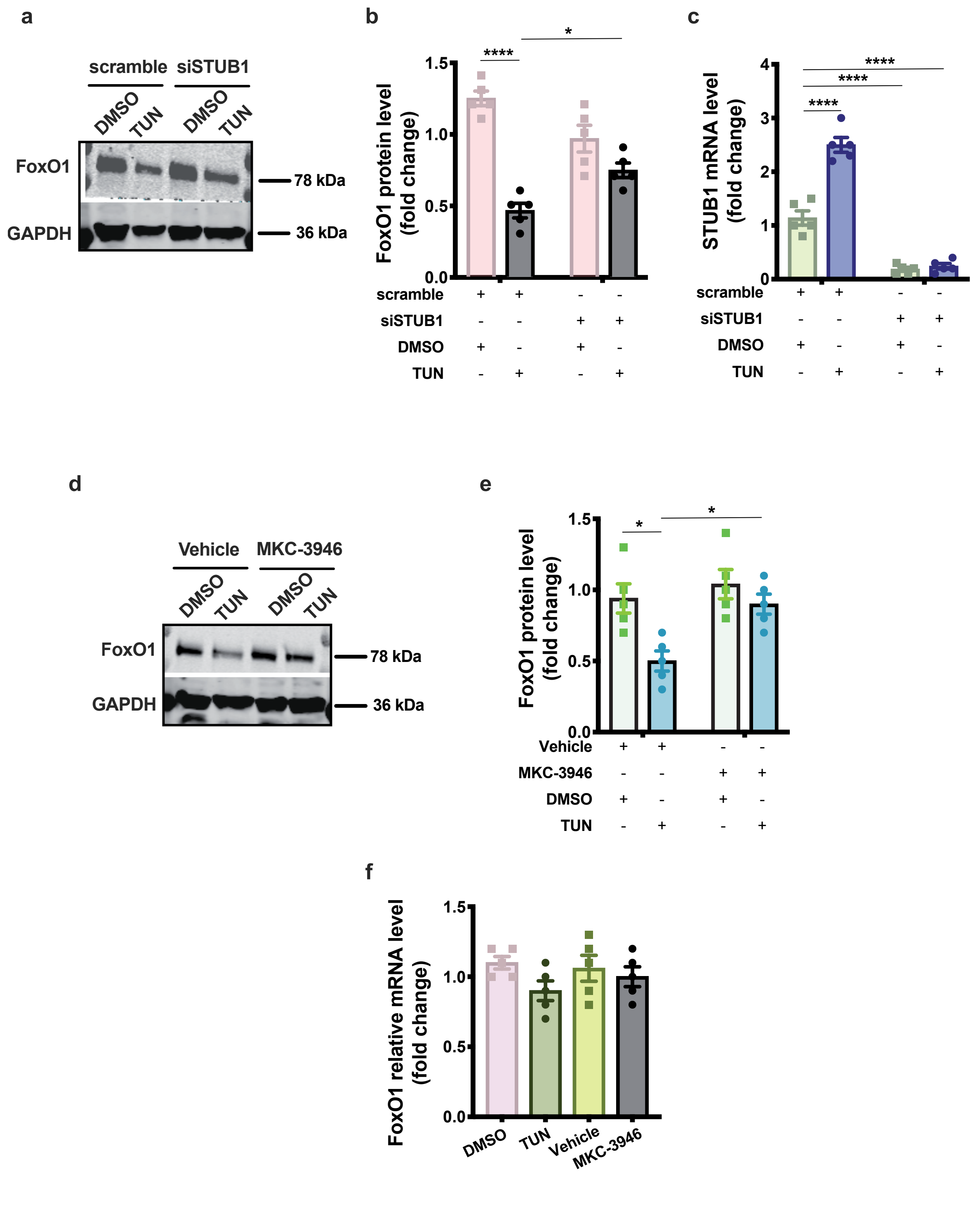

Supplementary Figure 9 | See next page for caption

Supplementary Figure 9 │ FoxO1 levels are decreased upon tunicamycin treatment in a Xbp1s- and STUB1-dependent manner. a, Representative immunoblot images of FoxO1 and GAPDH proteins from NRVMs treated with TUN or DMSO and transfected with siSTUB1 or scrambled siRNA control. Images are representative of 5 independent experiments. **b,** Densitometric analysis of FoxO1 protein band in the different experimental groups (n=5 biologically independent experiments). **c,** mRNA levels of *STUB1* in NRVMs treated with TUN or DMSO transfected with siSTUB1 or scrambled siRNA control (n=5 biologically independent experiments). **d,** Representative immunoblot images of FoxO1 and GAPDH proteins from NRVMs treated with TUN or DMSO and treated with MKC-3946 or Vehicle control. Images are representative of 5 independent experiments. **e,** Densitometric analysis of FoxO1 protein band in the different experimental groups (n=5 biologically independent experiments). **f,** mRNA levels of *FoxO1* in NRVMs treated with TUN or DMSO or MKC-3946 or vehicle control (n=5 biologically independent experiments). Results are presented as mean ± S.E.M. In b, c, e, f *p<0.05, ****p<0.0001, 1-way ANOVA plus Sidak's multiple comparisons test.

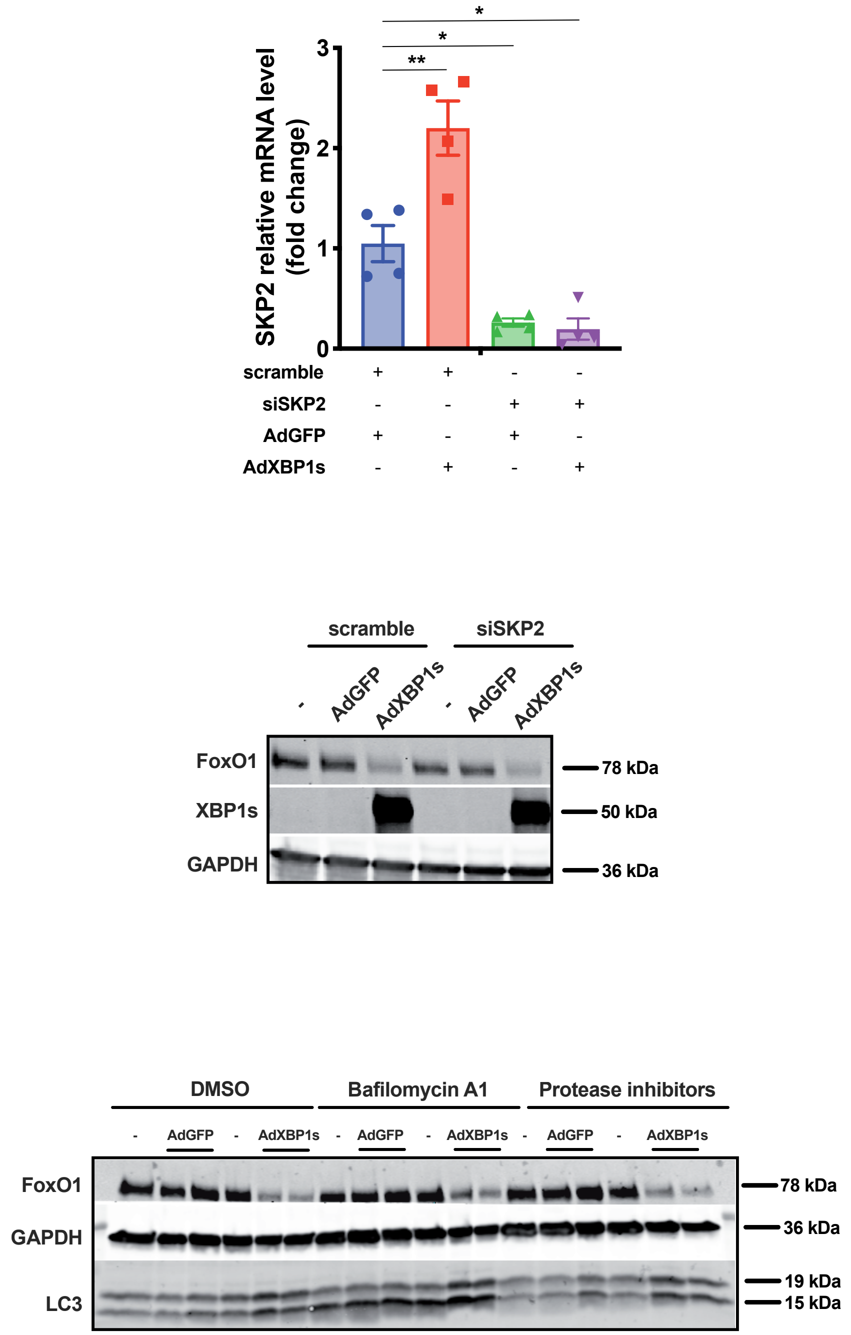

Supplementary Figure 10 | See next page for caption

Supplementary Figure 10 │ Xbp1s-dependent degradation of FoxO1 protein in cardiomyocytes is not mediated by SKP2. a, mRNA level of *SKP2* in NRVMs transfected with siSKP2 or scrambled siRNA control and transduced with AdGFP or AdXbp1s (n=4 biologically independent experiments). **b,** Representative immunoblot images of FoxO1, Xbp1s and GAPDH proteins from NRVMs transfected with siSKP2 or scrambled control and transduced with AdGFP, AdXbp1s or not transduced (-). Images are representative of 4 biologically independent experiments. **c,** Representative immunoblot images of FoxO1, GAPDH and LC3 proteins from NRVMs treated with DMSO, Bafilomycin A1 or cell-permeable protease inhibitors and transduced with AdGFP, AdXbp1s or not transduced (-). Images are representative of 3 biologically independent experiments. Results are presented as mean ± S.E.M. In a *$p<0.05$, **$p<0.005$, 1-way ANOVA plus Sidak's multiple comparisons test.

Supplementary Table 1. Echocardiographic parameters in the different experimental groups of mice

	CTR		HFpEF	
**(n=4-5/group)**	***FoxO1 F/F***	***FoxO1cKO***	***FoxO1 F/F***	***FoxO1 cKO***
Conscious				
HR (bpm)	656±16	645±21	651±23	650±16
LVID,d (mm)	2.8±0.1	2.7±0.2	2.9±0.4	2.9±0.3
LVID,s (mm)	1.1±0.2	1.1±0.3	1.2±0.1	1.2±0.1
IVS,d (mm)	1.0±0.2	1.1±0.1	1.3±0.2	1.3±0.1
LVPW,d (mm)	1.0±0.1	0.9±0.2	1.1±0.3	1.1±0.2
LVFS (%)	61.3±3.2	59.3±2.6	58.6±1.5	58.6±1.4
LV mass (mg)	95.4±6.7	81.9±5.3	131.3±5.4*	131.3±6.4*
LVEF (%)	92.1±0.2	90.5±1.1	91.2±0.9	91.2±1.1
Unconscious				
HR (bpm)	433±13	444±10	4392±11	435±15
Mitral E/A	1.2±0.06	1.1±0.11	1.9±0.10*	1.4±0.12#
Mitral E/E'	30.1±0.6	28.5±1.0	42.4±4.3*	26.8±2.0#
**(n=5/group)**	***Xbp1s WT***	***Xbp1s TG***	***Xbp1s WT***	***Xbp1s TG***
Conscious				
HR (bpm)	666±19	678±14	6770±13	665±17
LVID,d (mm)	2.7±0.3	2.6±0.2	2.8±0.3	2.9±0.1
LVID,s (mm)	1.1±0.2	1.0±0.1	1.2±0.3	1.2±0.2
IVS,d (mm)	1.0±0.1	1.0±0.1	1.2±0.2	1.1±0.1
LVPW,d (mm)	0.9±0.2	1.0±0.3	1.1±0.1	1.1±0.2
LVFS (%)	59.3±2.4	61.5±3.1	57.1±2.3	57.1±2.3
LV mass (mg)	81.9±6.4	84.1±8.1	116.8±4.4*	108.7±5.5*
LVEF (%)	91.7±1.0	92.8±0.7	91.2±1.2	91.5±1.1
Unconscious				
HR (bpm)	441±14	439±15	440±11	437±12
Mitral E/A	1.2±0.08	1.2±0.13	2.5±0.09*	1.8±0.08#
Mitral E/E'	28.7±1.0	26.3±0.8	43.9±2.1*	30.7±1.3#

Data are expressed as mean ± S.E.M

Abbreviations used: HR, heart rate; **LVID,d,** left ventricular internal diastolic diameter; **LVID,s**, left ventricular internal systolic diameter; **IVS,d**, end-diastolic interventricular septal wall thickness; **LVPW,d**, left ventricular end-diastolic posterior wall; **LVFS**, left ventricular **LVEF**, left ventricular ejection fraction; **E**, peak Doppler blood inflow velocity across mitral valve during early diastole; **A**, peak Doppler blood inflow velocity across mitral valve during late diastole; **E'**, peak tissue Doppler of myocardial relaxation velocity at mitral valve annulus during early diastole. *p<0.01 vs. CTR F/F or WT; #p<0.001 vs. HFpEF F/F or WT.

Supplementary Table 2. List of primers used in the study

Gene	Species	Purpose	Forward	Reverse
Xbp1s	mouse/rat	qPCR	GGTCTGCTGAGTCCGCAGCAGG	GAAAGGGAGGCTGGTAAGGAAC
FASN	mouse	qPCR	ATCTCTCCAAGTTCGACGCC	GTTCGTTCCTCGGAGTGAGG
SERBP1	mouse	qPCR	GGACACAGCGGTTTTGAACG	CTCAGGAGAGTTGGCACCTG
CPT1b	mouse	qPCR	CCCTCATGGTGAACAGCAACT	GCCATGACCGGCTTGATCTC
FoxO1	mouse	qPCR	TCAAGGATAAGGGCGACAGC	CCTCCCTCTGGATTGAGCATC
Pdk4	mouse	qPCR	GCTTGCCAATTTCTCGTCTC	CTTCTCCTTCGCCAGGTTCT
P21	mouse	qPCR	GGTGTCAGAGTCTAGGGGAA	AGACAACGGCACACTTTGCT
ACOX1	mouse	qPCR	CTACGCCCAGACGGAGATG	GAAGTCTTCCCAAGCCCC
ACAB	mouse	qPCR	CACCCAACTCTGAAGGGGAC	TCCACAGCAATCACTCCCAC
PPARγ	mouse	qPCR	CGGGCTGAGAAGTCACGTT	TGTGTCAACCATGGTAATTTCAGT
ATGL	mouse	qPCR	CCACTCACATCTACGGAGCC	GATGCAGAGGACCCAGGAAC
CD36	mouse	qPCR	TGTGGAGCAACTGGTGGATG	CGTGGCCCGGTTCTAATTCA
SLC27A1	mouse	qPCR	ACTCTGCAAAGGGCTCATCC	CATCAGATCCCTGGCCCTTG
GOT2	mouse	qPCR	GGAAGGAGATAGCGTCCGTG	AGAGGCAGACATTGATGCCC
ANP	mouse	qPCR	CTGGGACCCCTCCGATAGAT	TTCGGTACCGGAAGCTGTTG
BNP	mouse	qPCR	GAGTCCTTCGGTCTCAAGGC	CAACTTCAGTGCGTTACAGC
βMHC	mouse	qPCR	AAGCAGCAGTTGGATGAGCG	CCTCGATGCGTGCCTGAAGC
RCAN1.4	mouse	qPCR	CCCGTGAAAAAGCAGAATGC	TCCTTGTCATATGTTCTGAAGAGGG
STUB1	mouse/rat	qPCR	GGCGGAGCCTTGGTCTGA	GGCGGAGCCTTGGTCTGA
FoxO1	rat	qPCR	GGATAAGGGCGACAGCAACA	TCTTGCCTCCCTCTGGATTG
pdk4	rat	qPCR	TTTCTGACCGAGGTGGCGGT	AACCAGCCAAAGGGGCATTCCG
p21	rat	qPCR	CTTGTGATATGTACCAGCCACAG	AAAGTTCCACCGTTCTCGGG
SKP2	rat	qPCR	GTGTCAAACCTCCACGGGAT	TCTGCGCAAGAGTAGTGACA
18S	mouse/rat	qPCR	AAACGGCTACCACATCCAAG	CCTCCAATGGATCCTCGTTA

UNCROPPED SCANS

Figure 1C

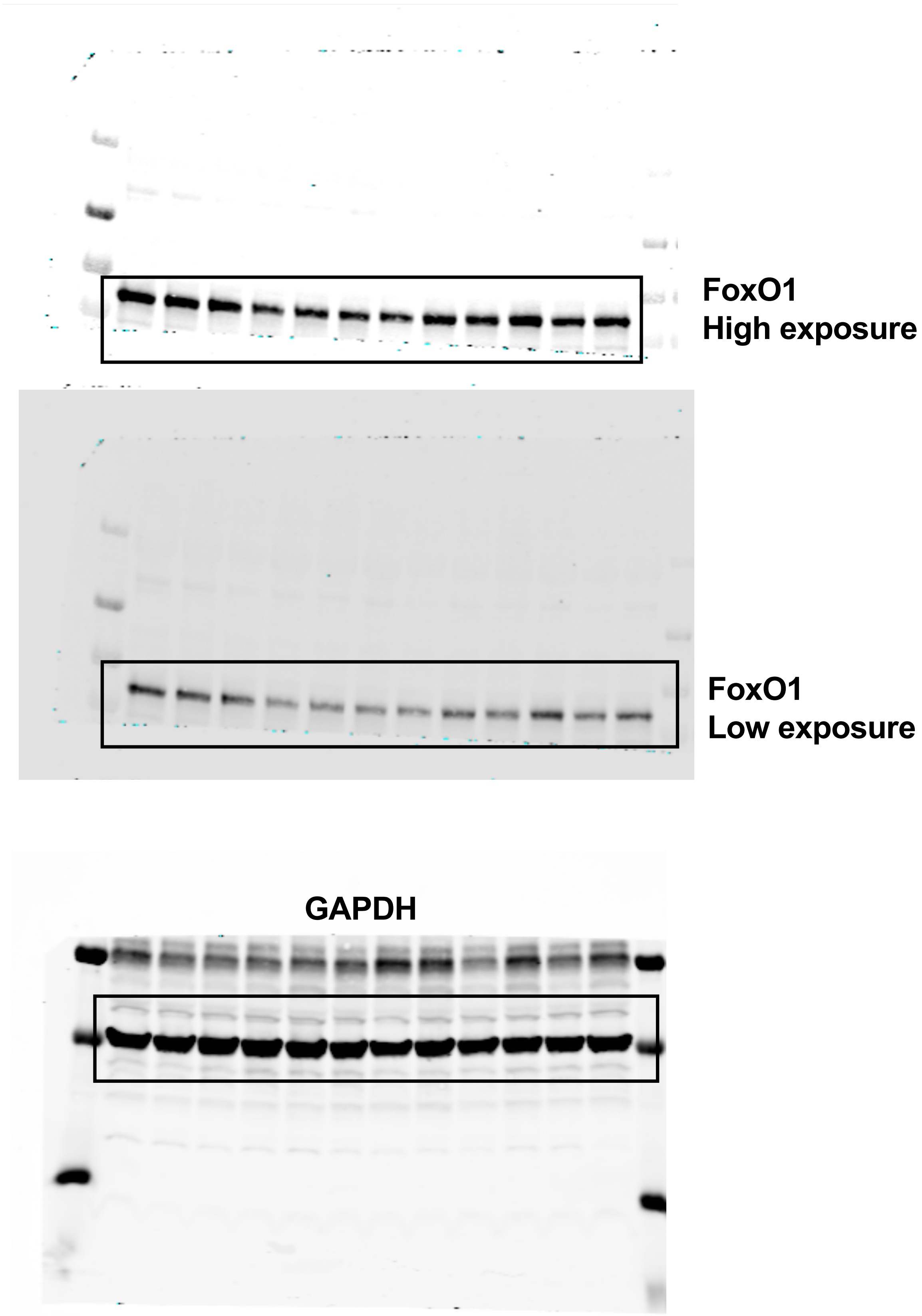

Figure 1E

FoxO1

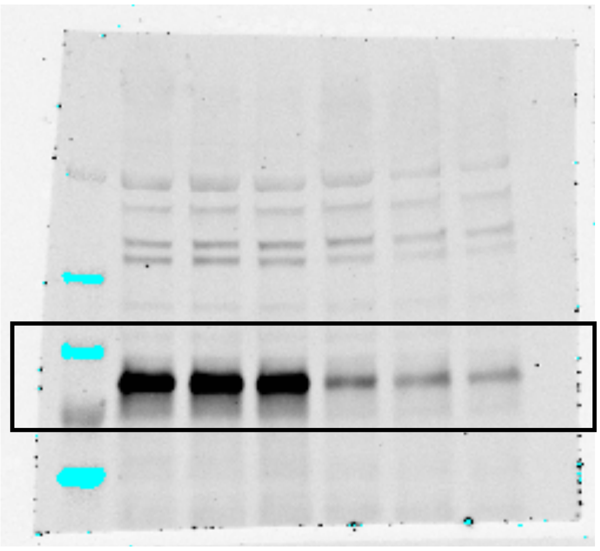

GAPDH

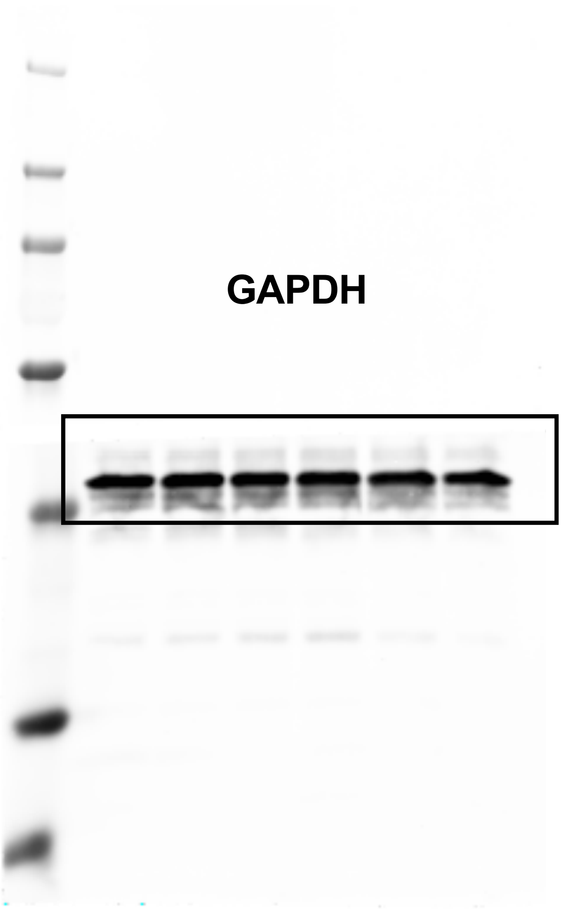

Figure 1G

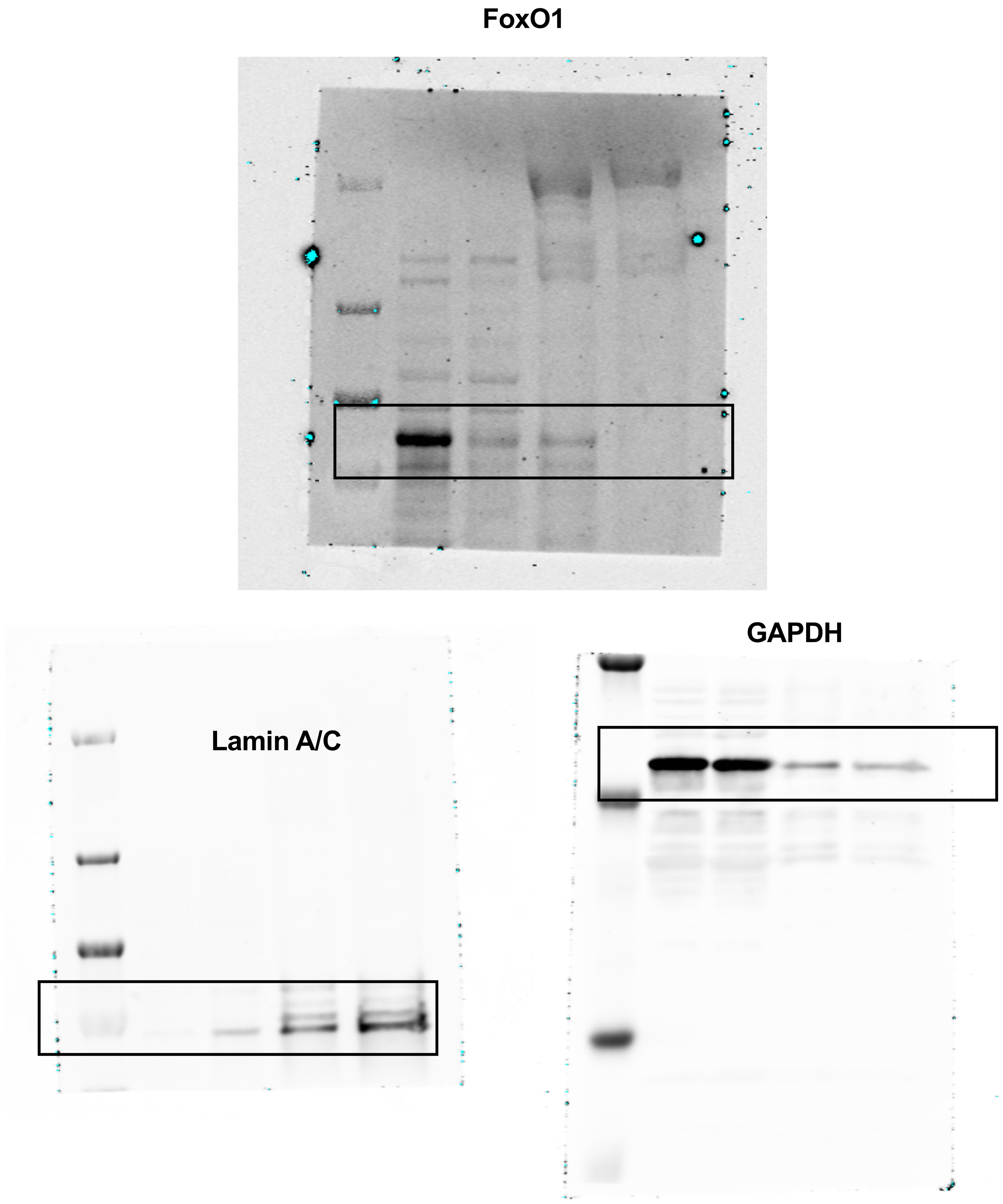

Figure 1I

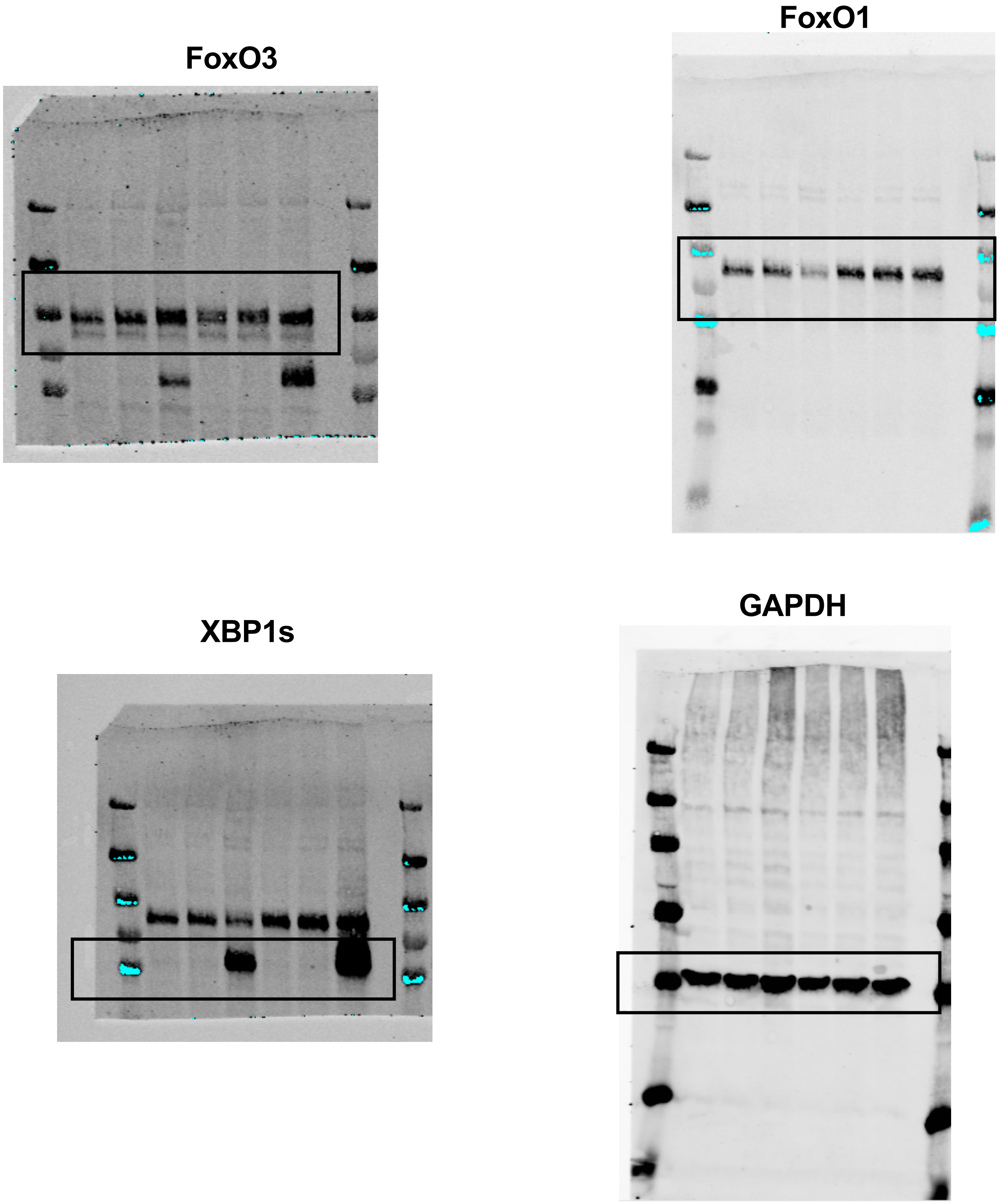

Figure 2A

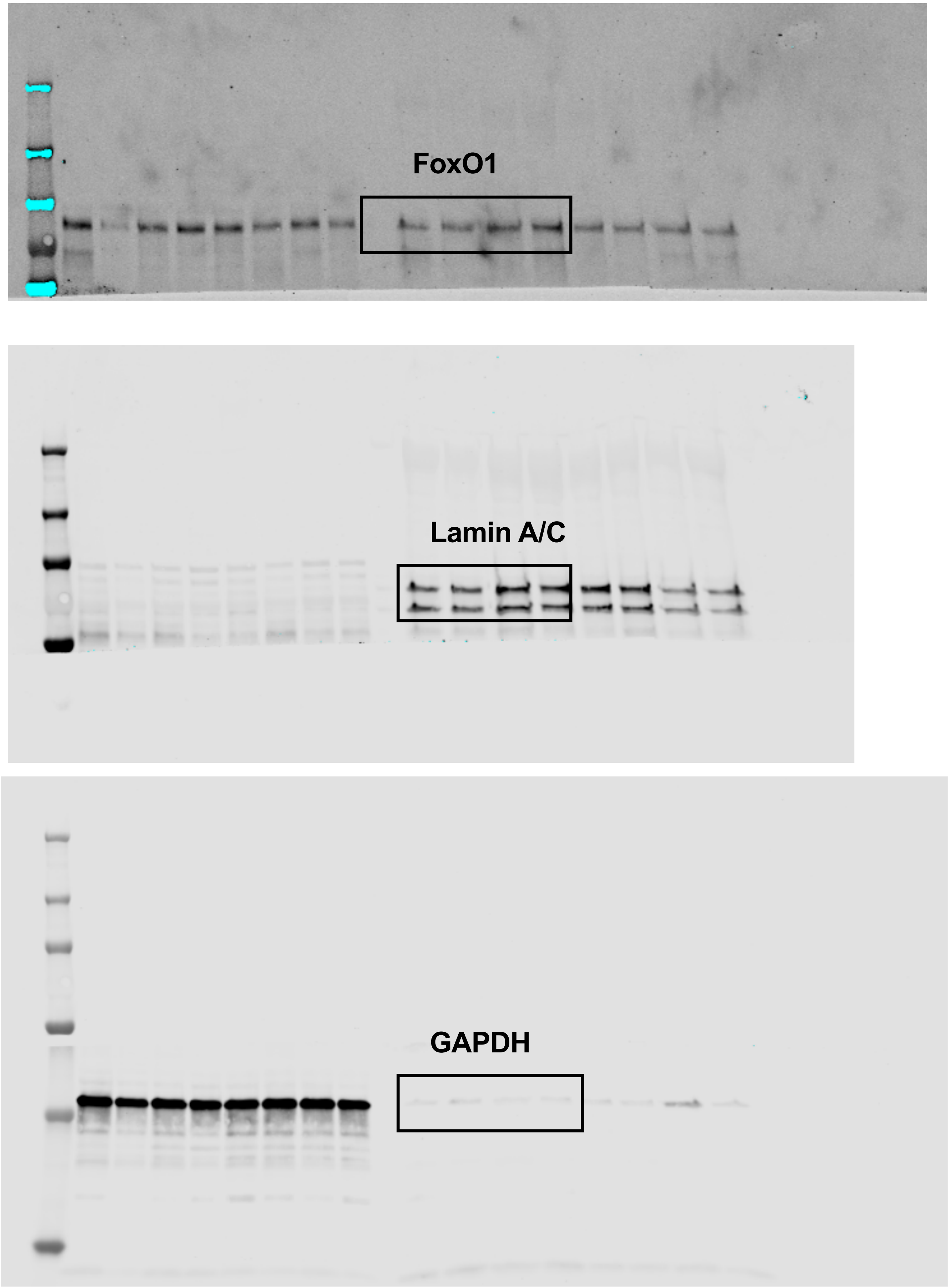

Figure 2C

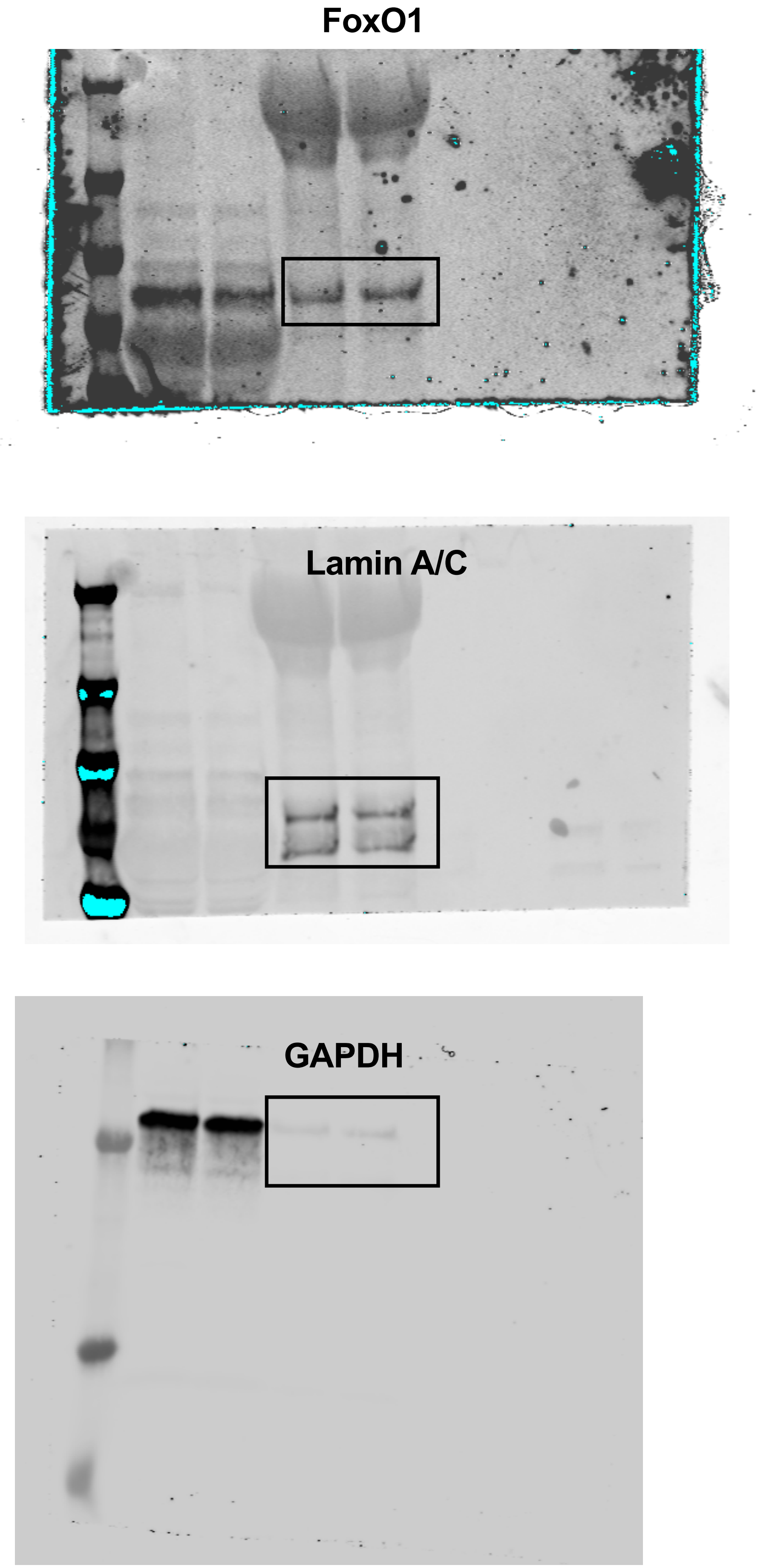

Figure 4C

Agarose Gel CHIP Assay

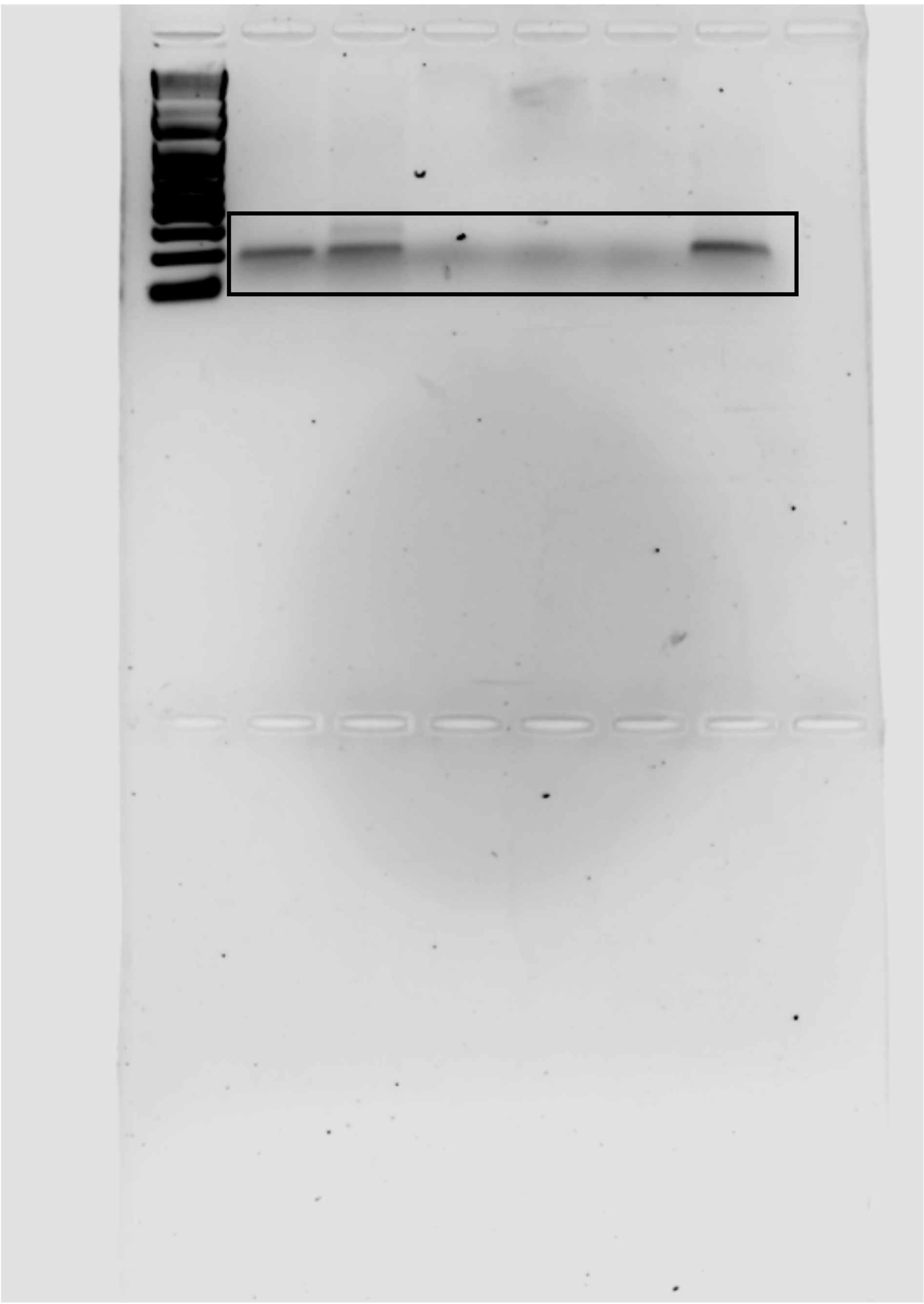

Figure 4F

XBP1s

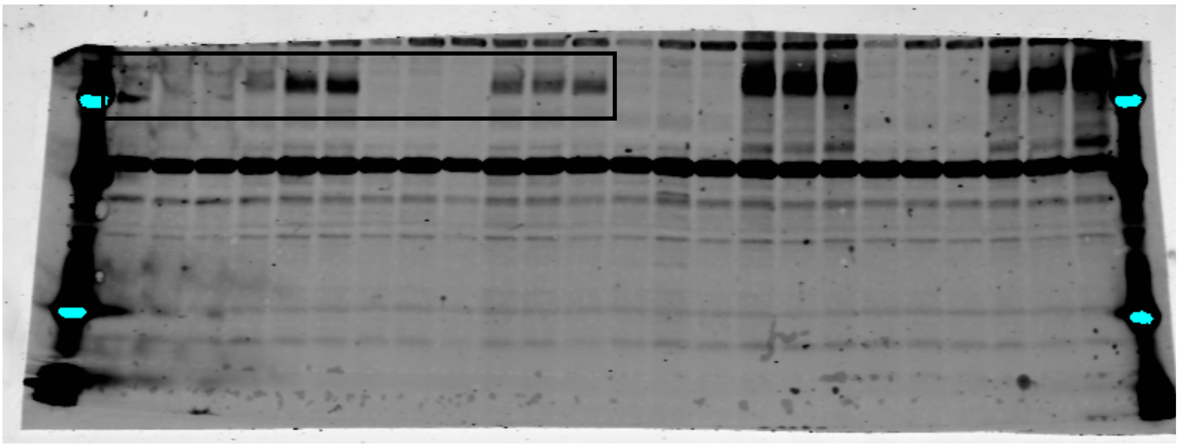

STUB1

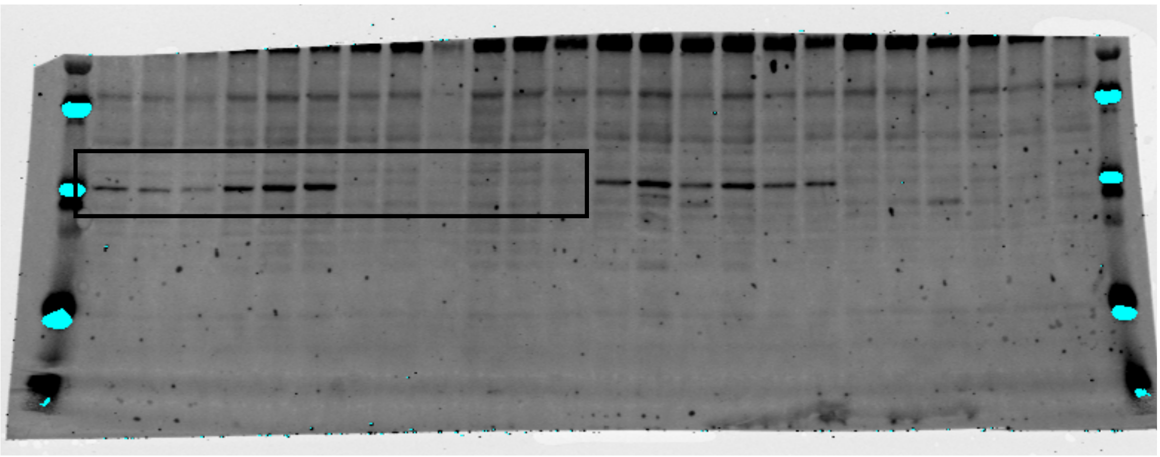

GAPDH

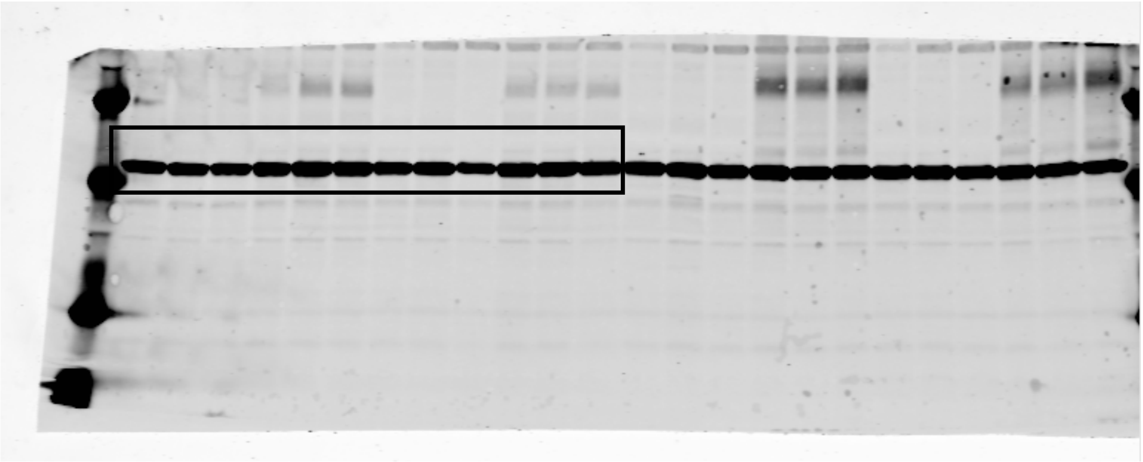

Figure 4H

XBP1s high exp

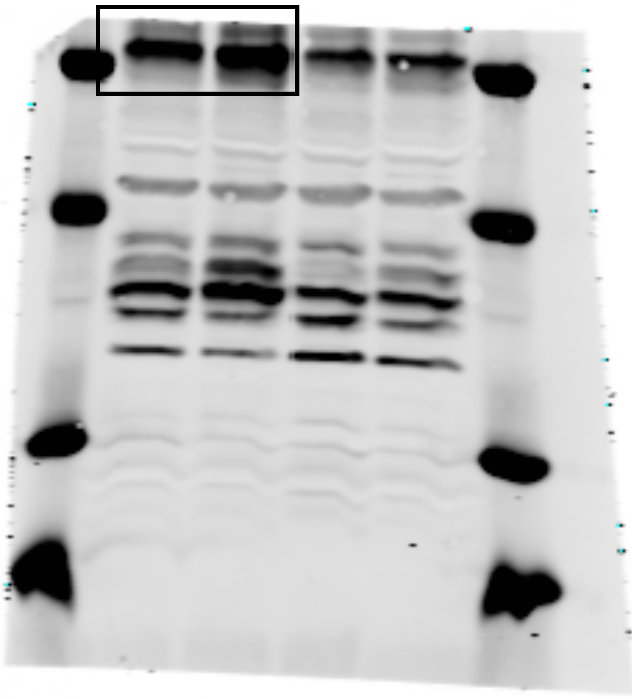

XBP1s low exp

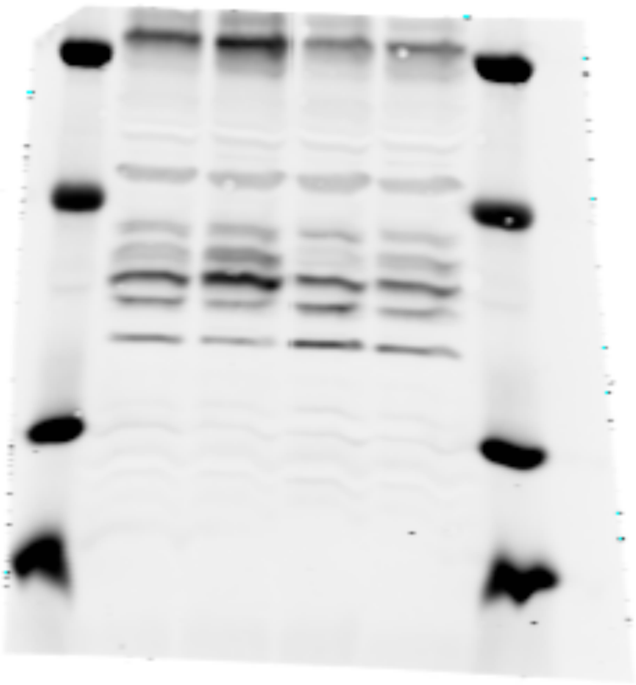

STUB1

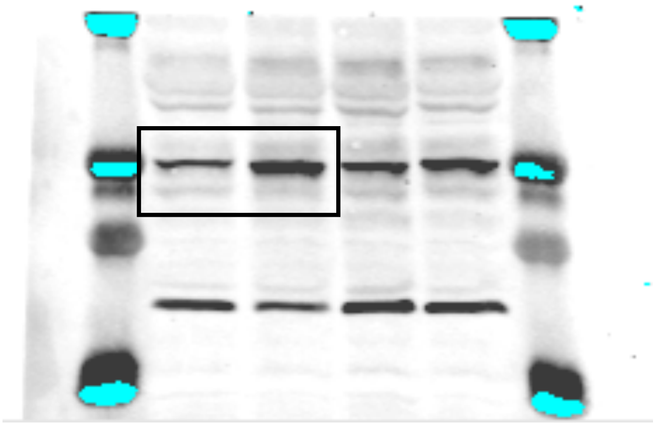

GAPDH

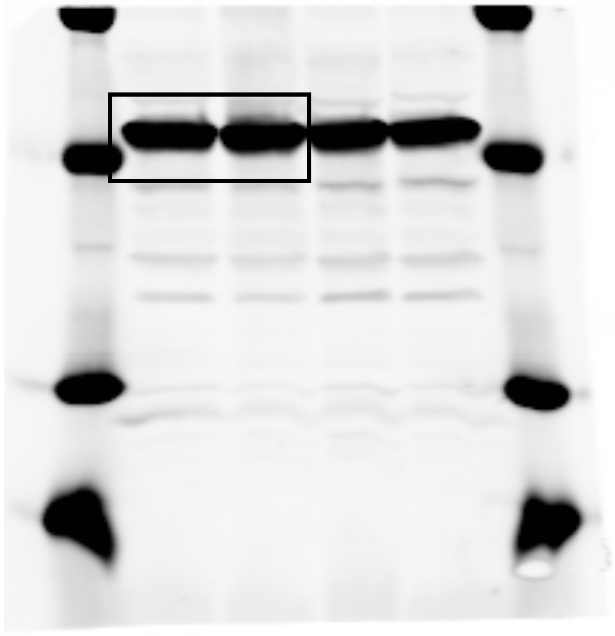

Figure 5A

FoxO1 low exposure

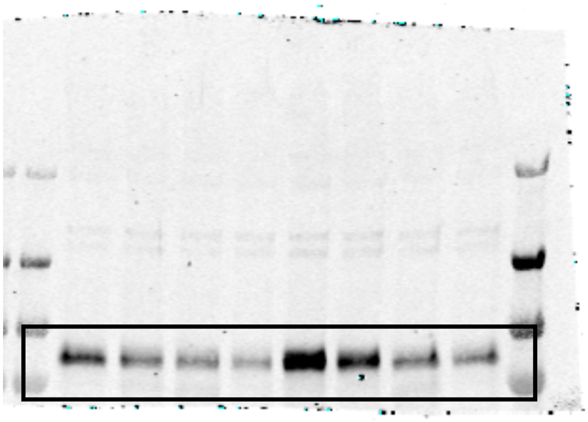

FoxO1 high exposure

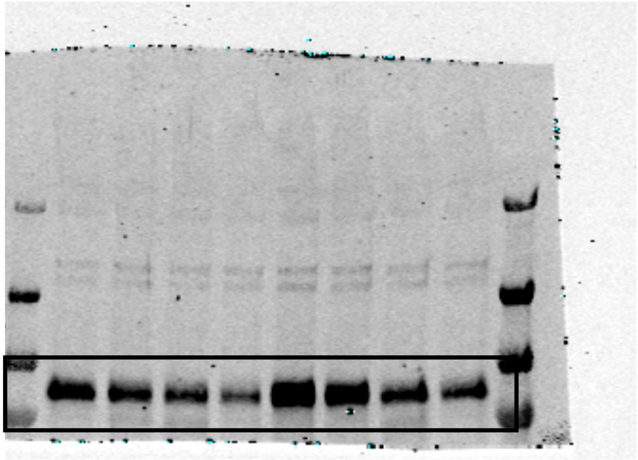

STUB1

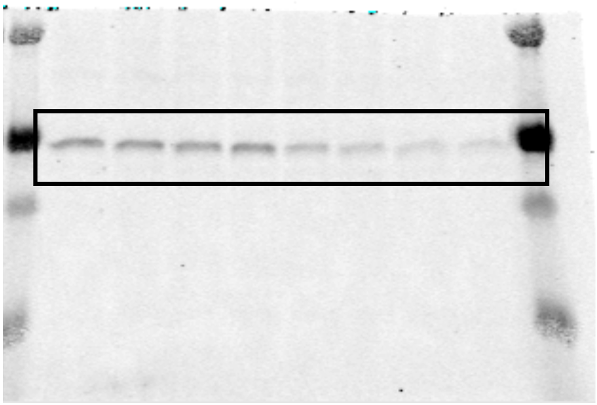

GAPDH

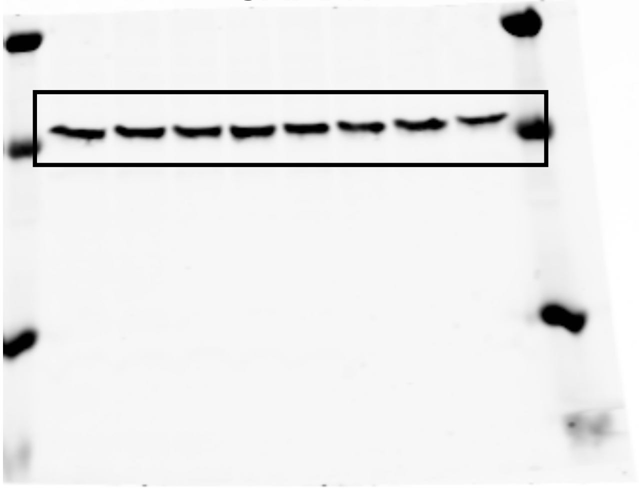

Figure 5D

FoxO1

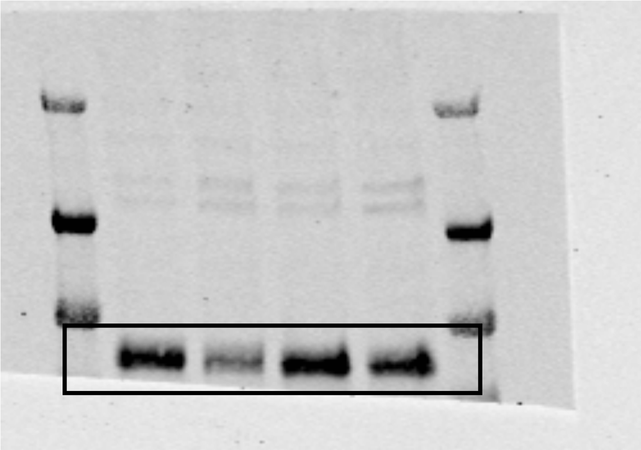

XBP1s

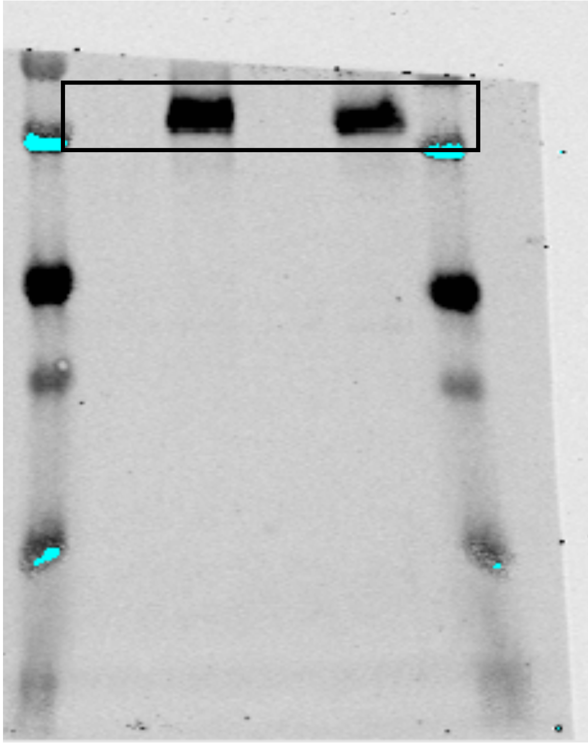

STUB1

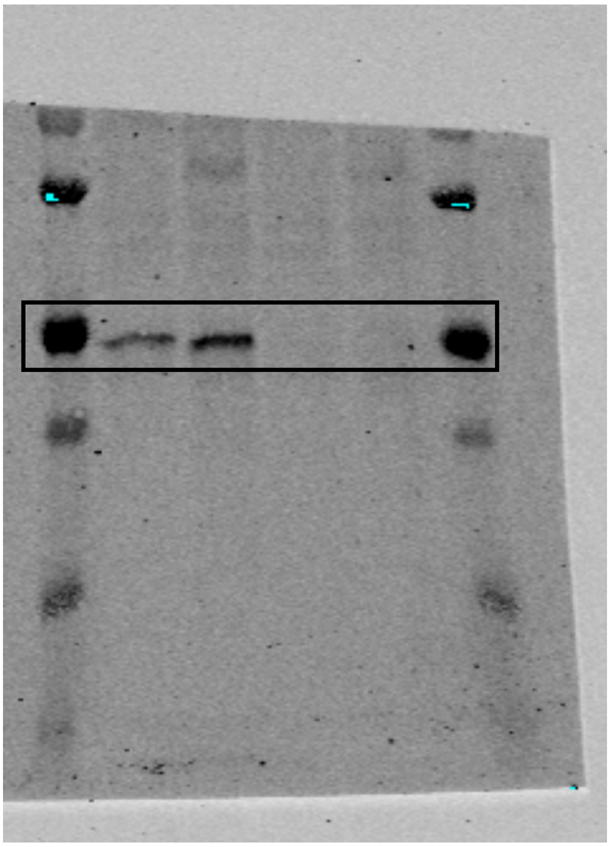

GAPDH

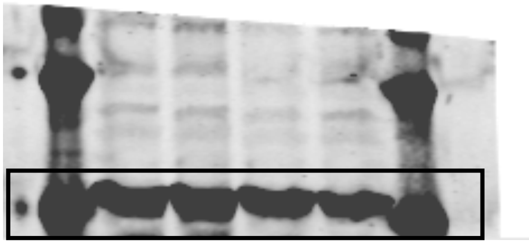

Suppl. Figure 2H

XBP1s

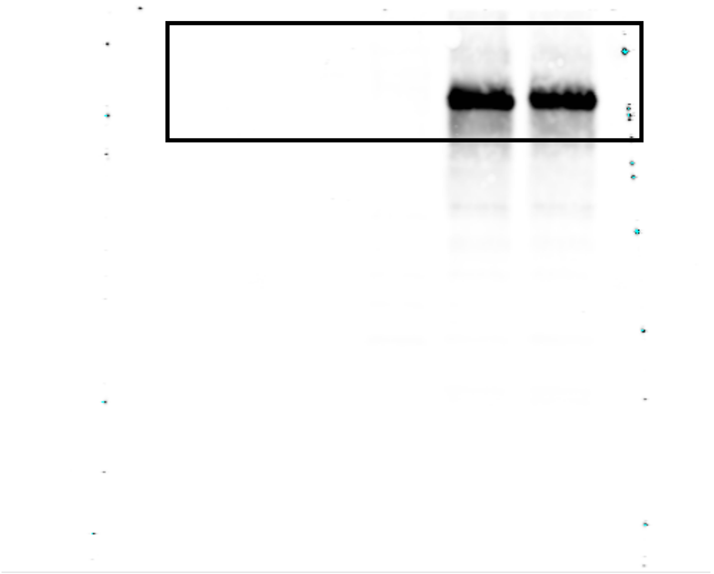

GFP

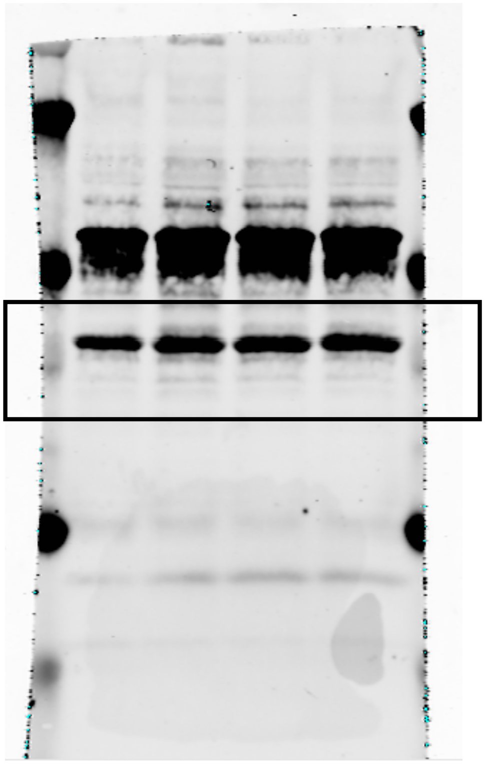

GAPDH

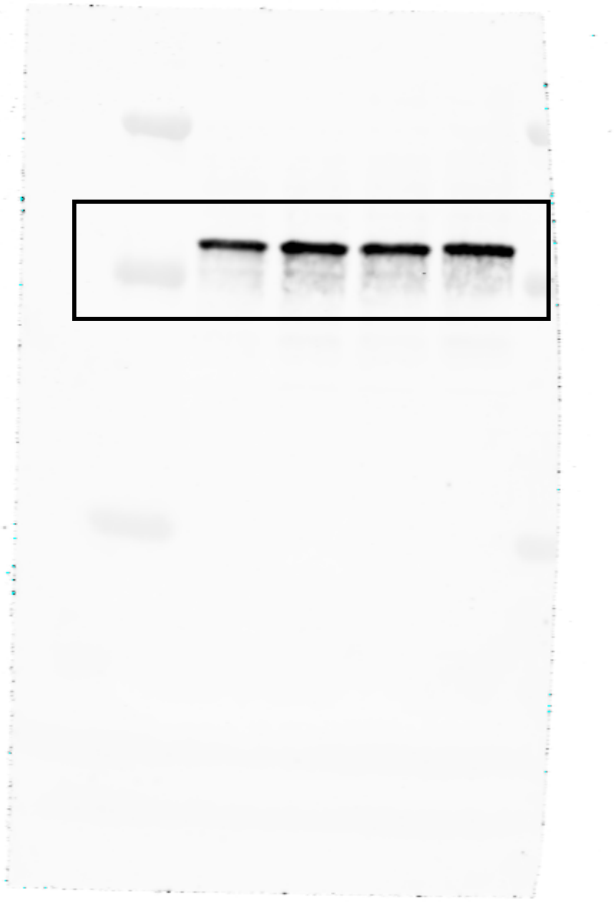

Suppl. Figure 3 (I)

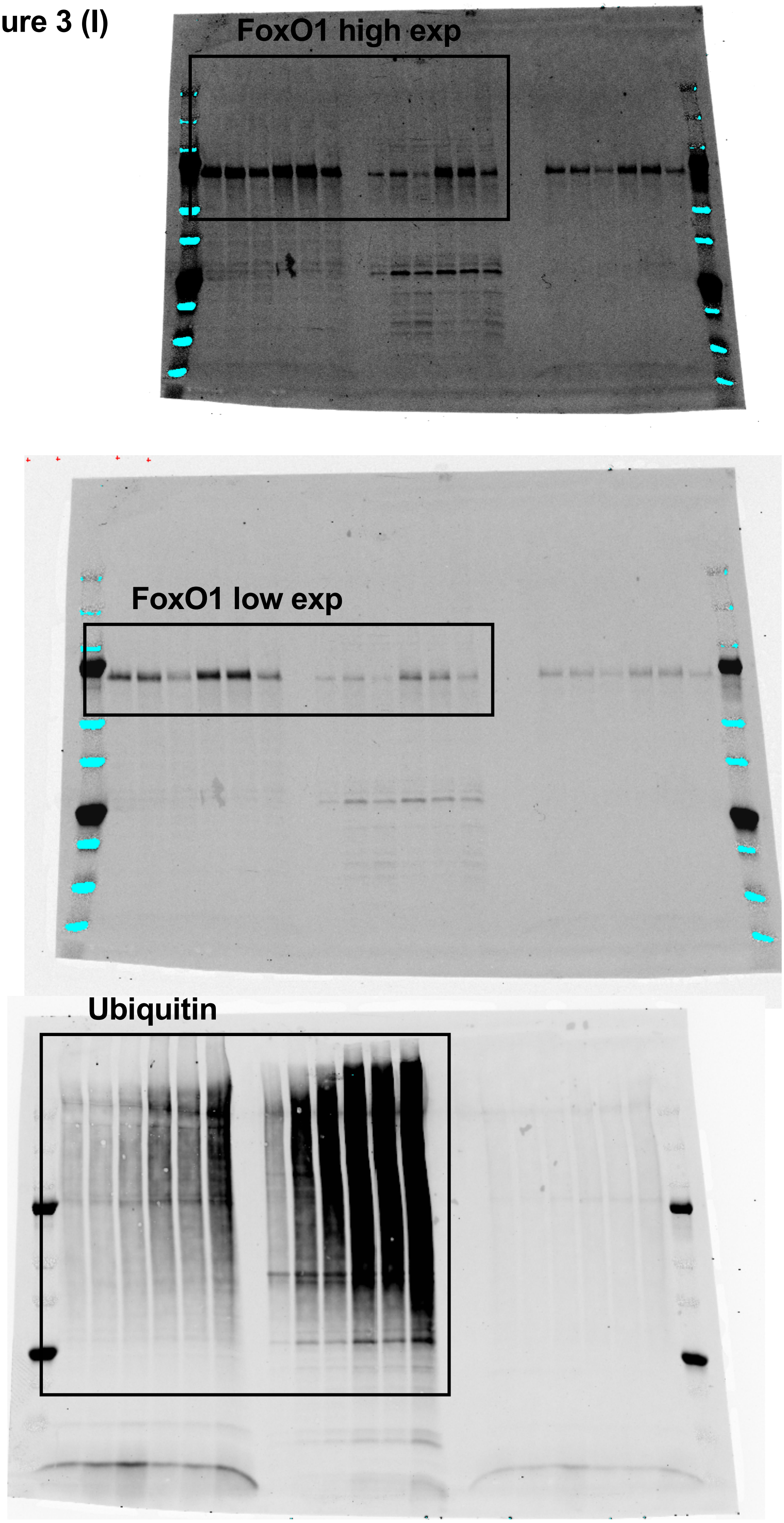

Suppl. Figure 3 (II)

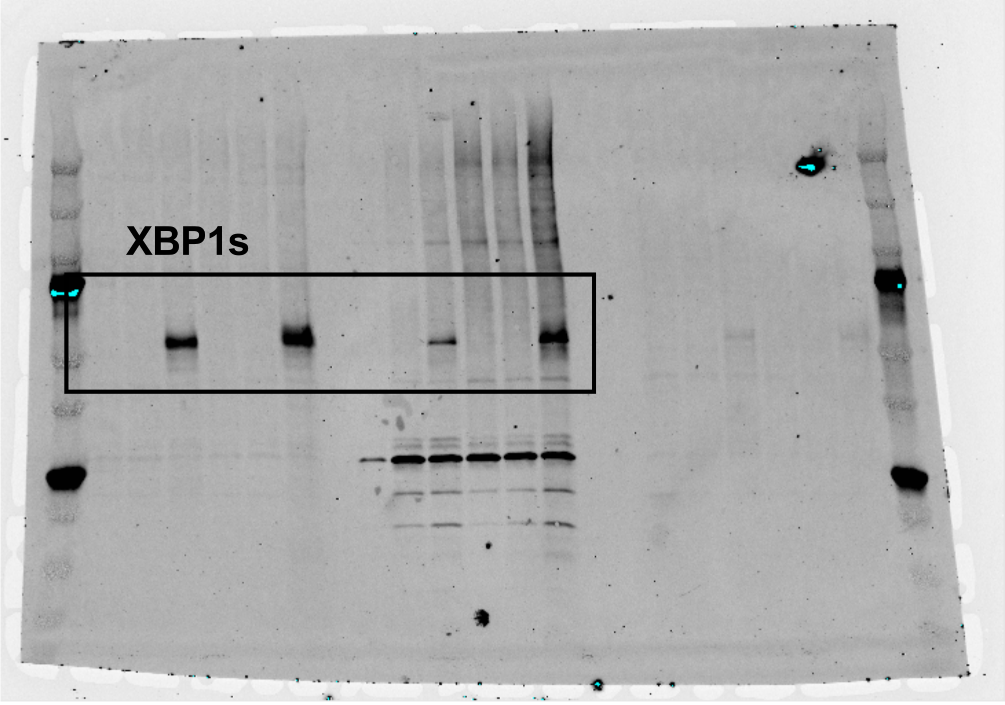

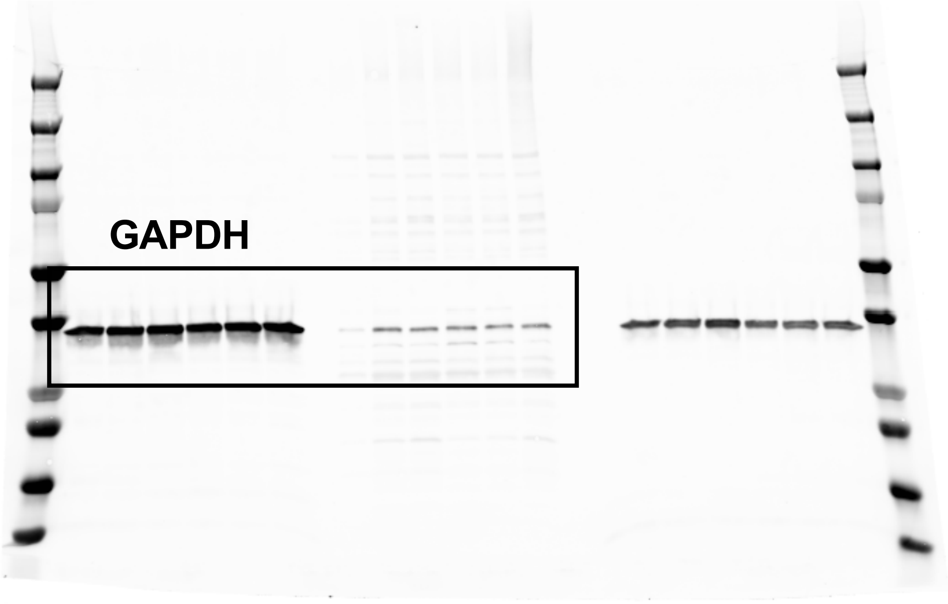

Suppl. Figure 6B

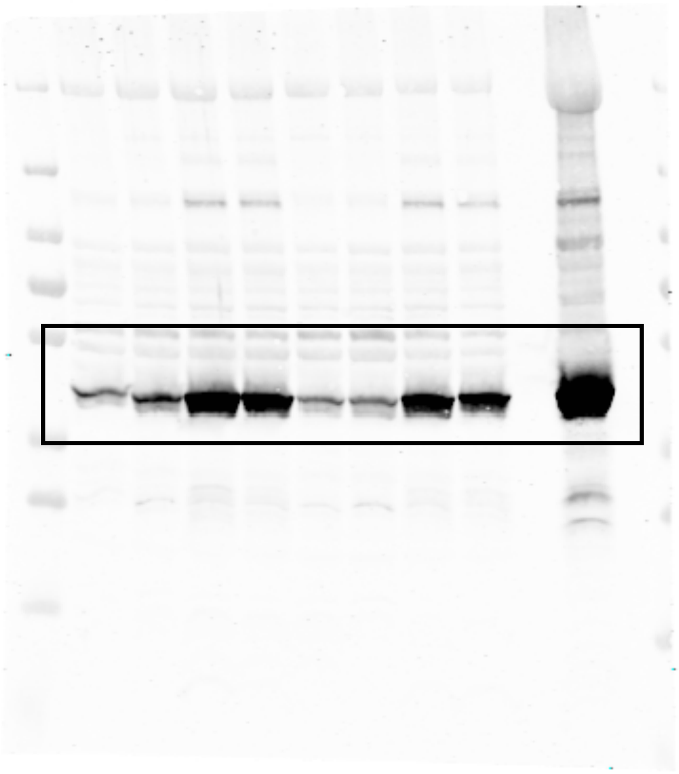

PDK4 high exposure

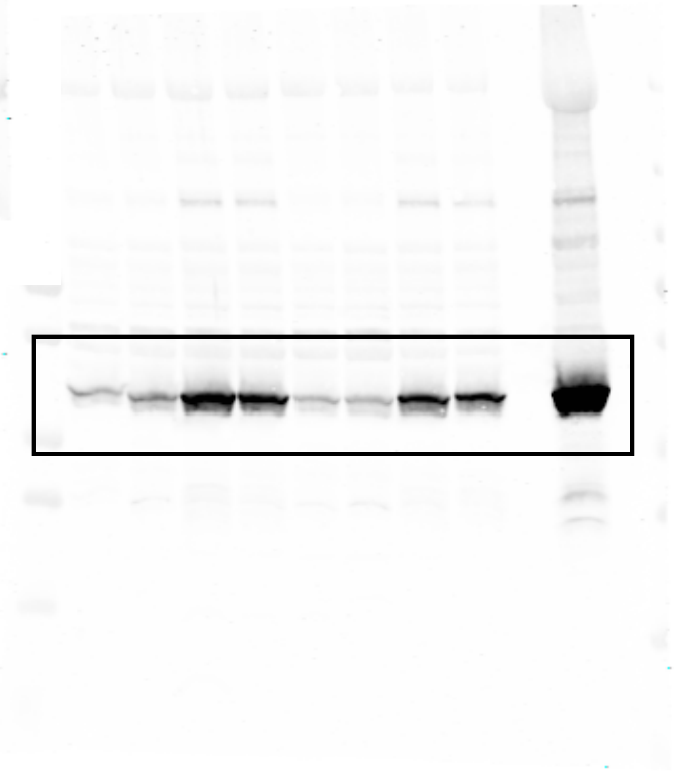

PDK4 low exposure

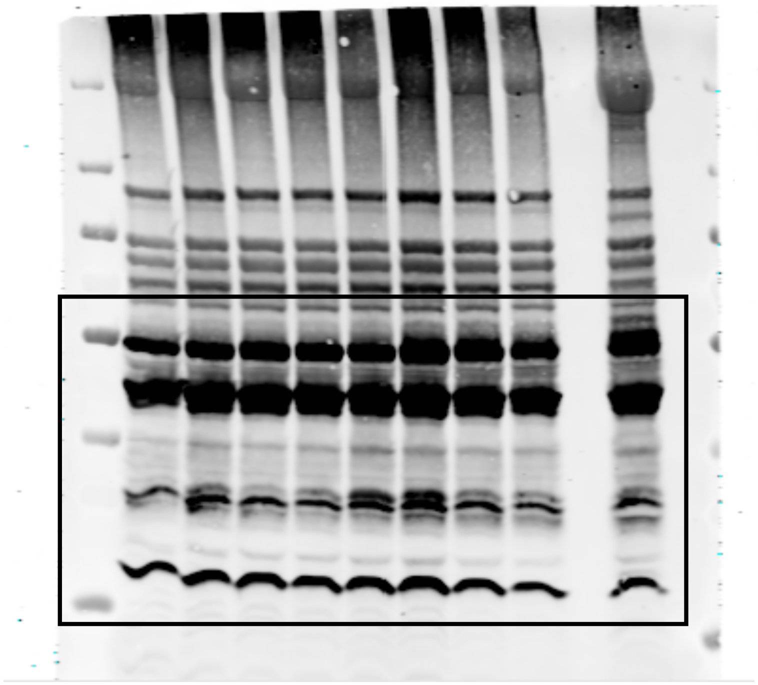

OXPHOS

Suppl. Figure 8A (I)

Ubiquitin

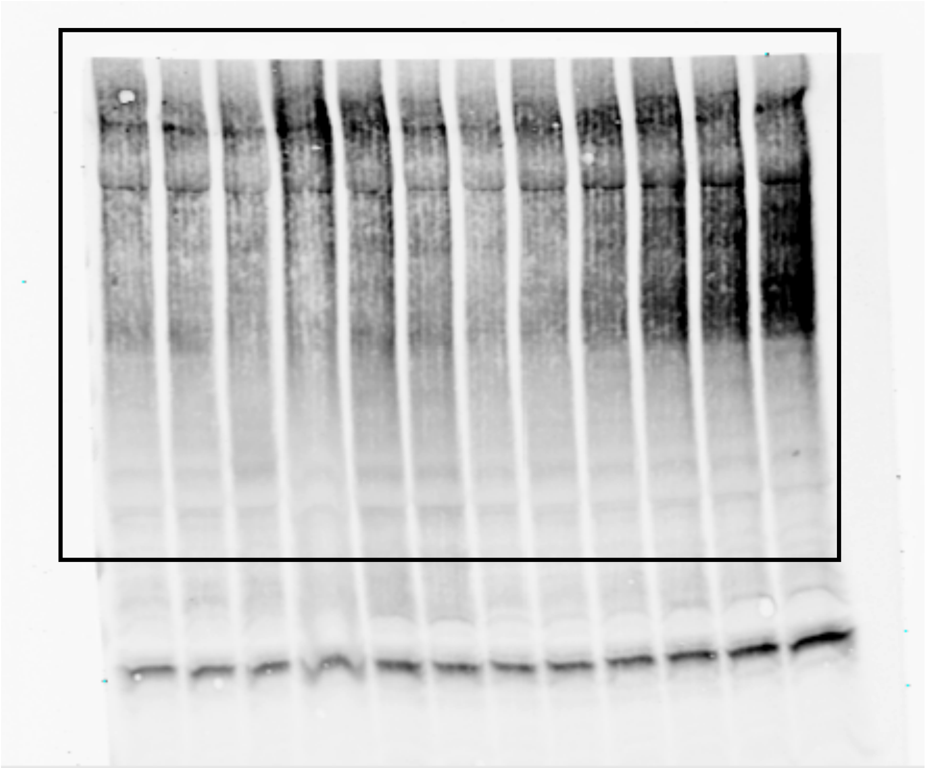

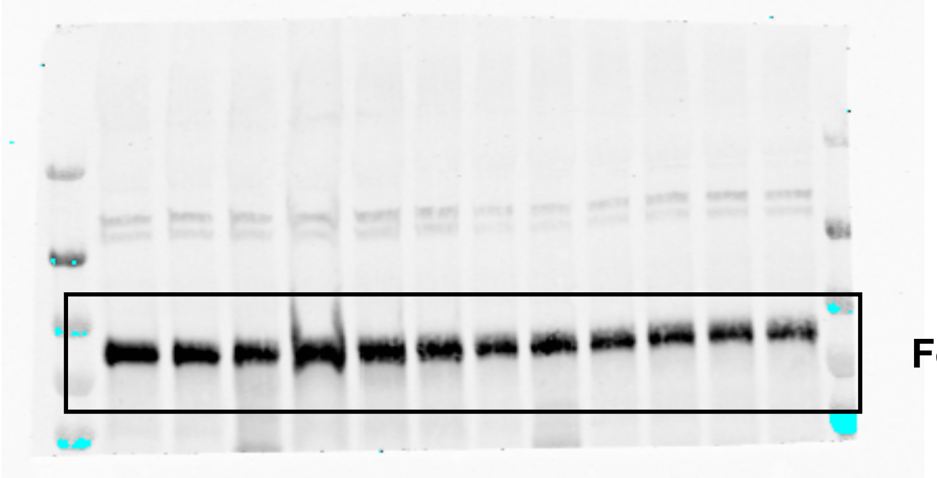

FoxO1 high exposure

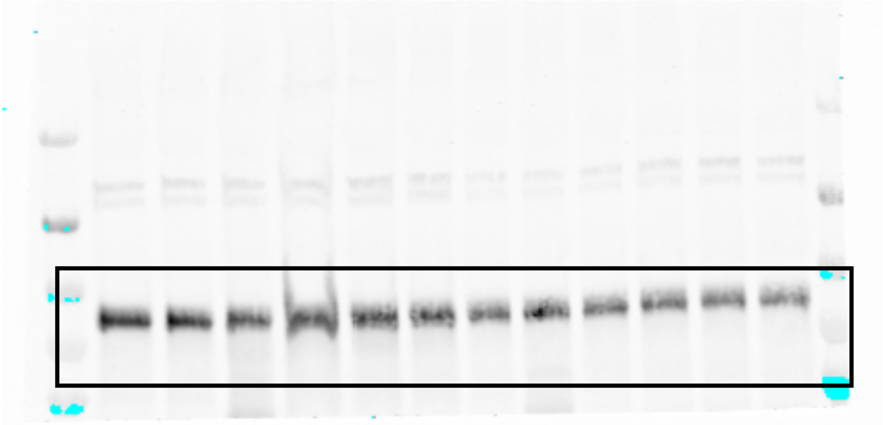

FoxO1 low exposure

Suppl. Figure 8A (I)

STUB1

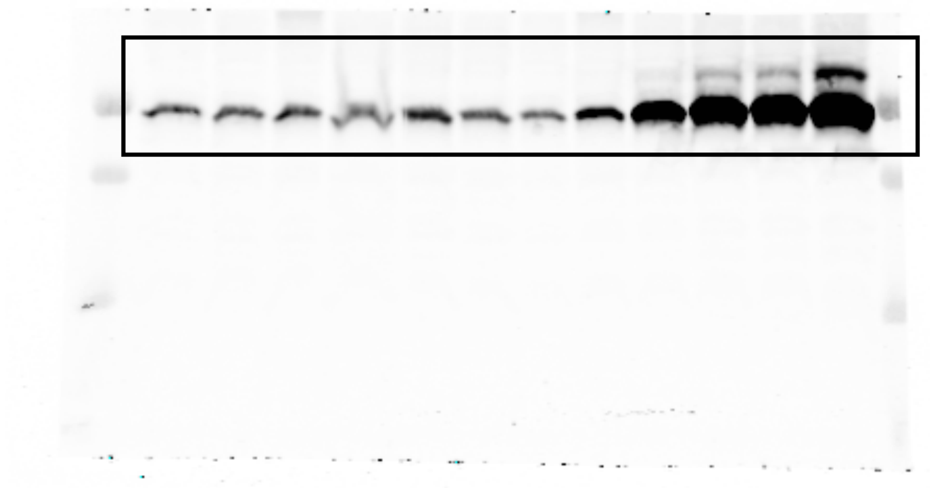

GAPDH

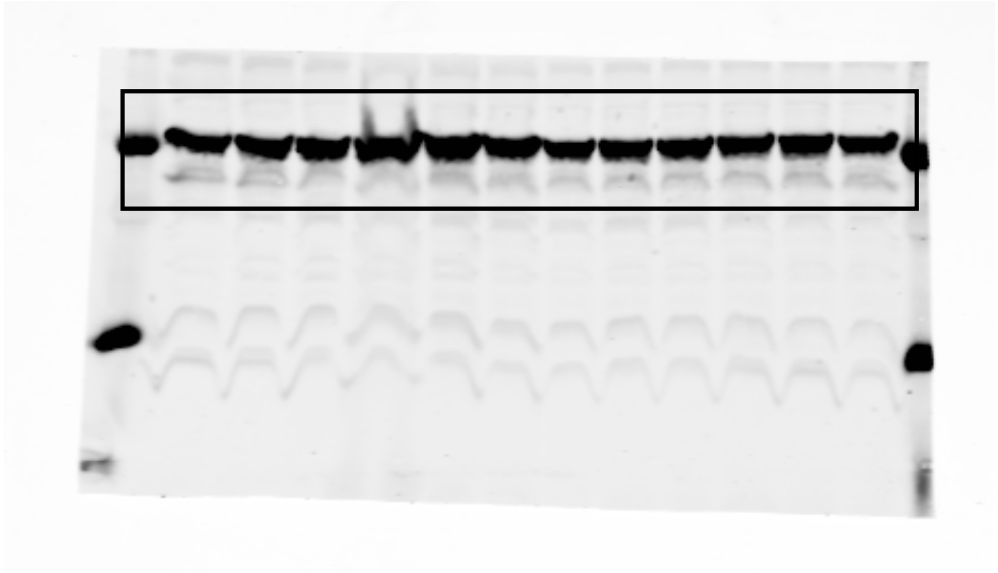

Suppl. Figure 9A

GAPDH

FoxO1

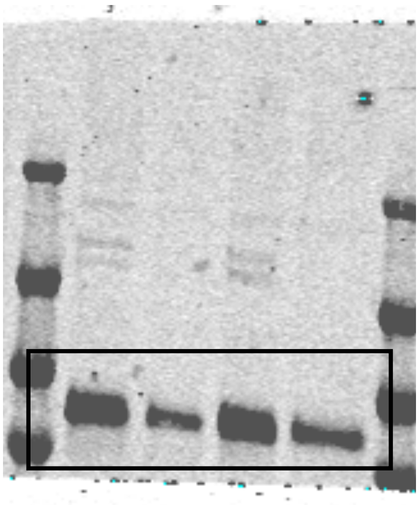

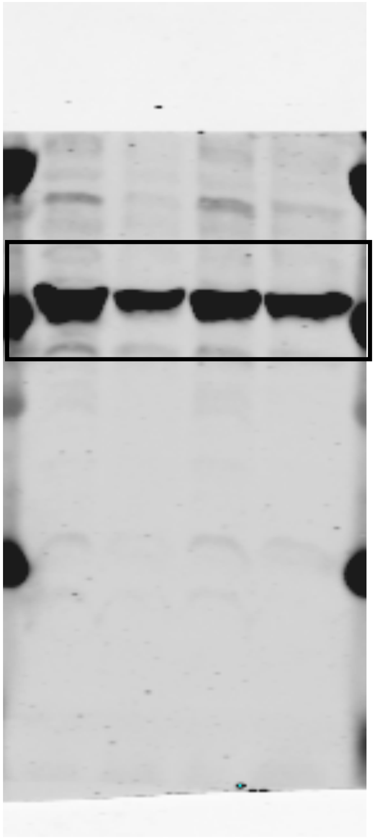

Suppl. Figure 9D

FoxO1

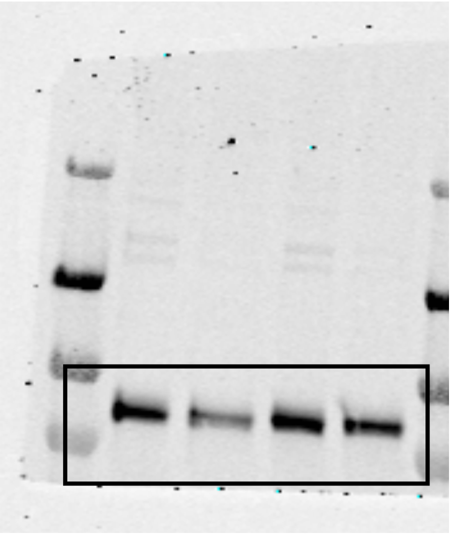

GAPDH

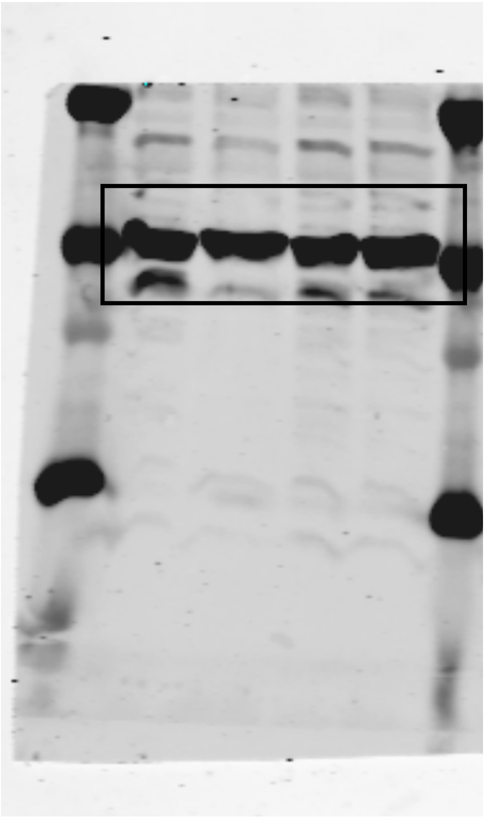

Suppl. Figure 10B

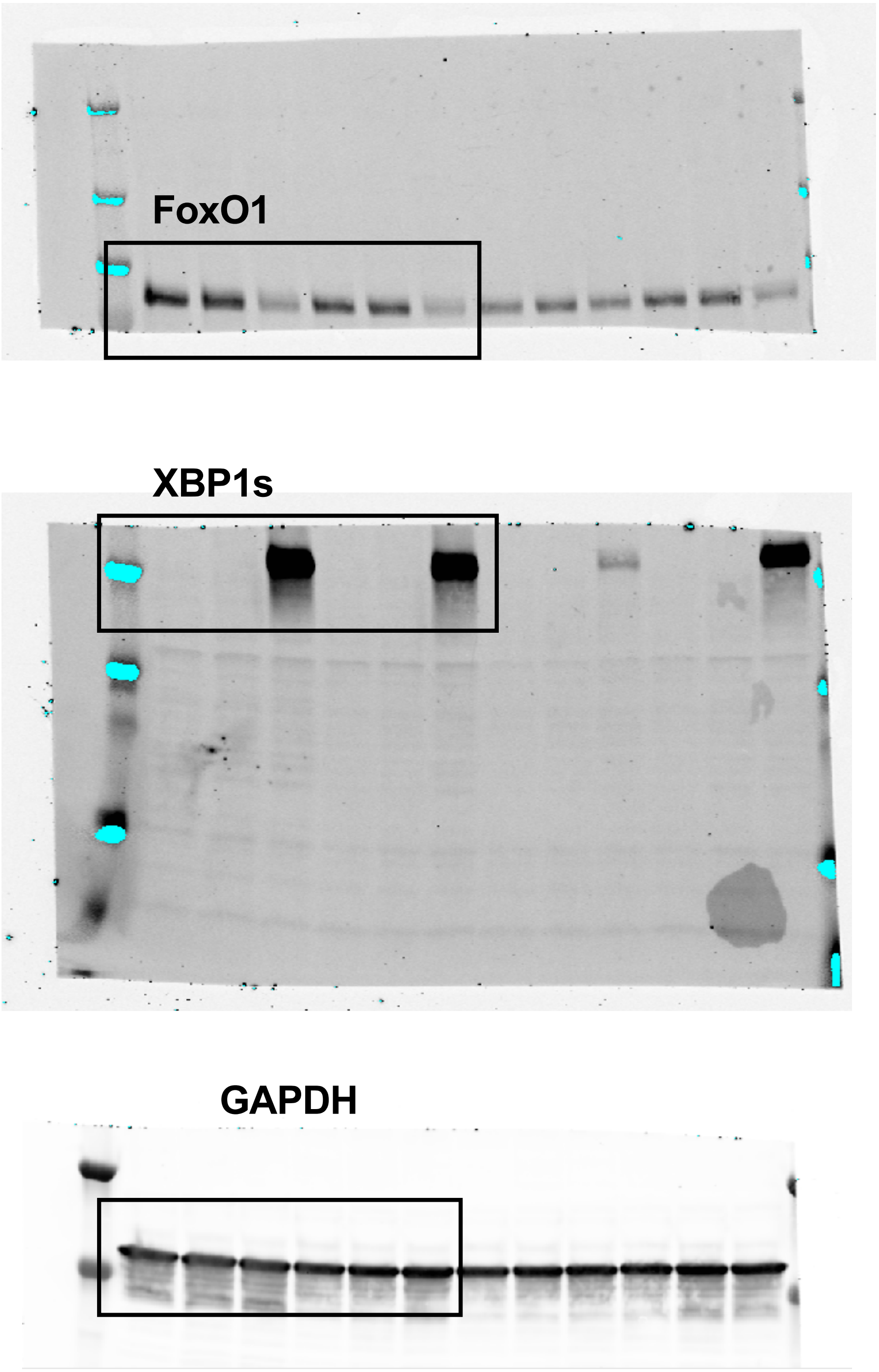

Suppl. Figure 10C

FoxO1

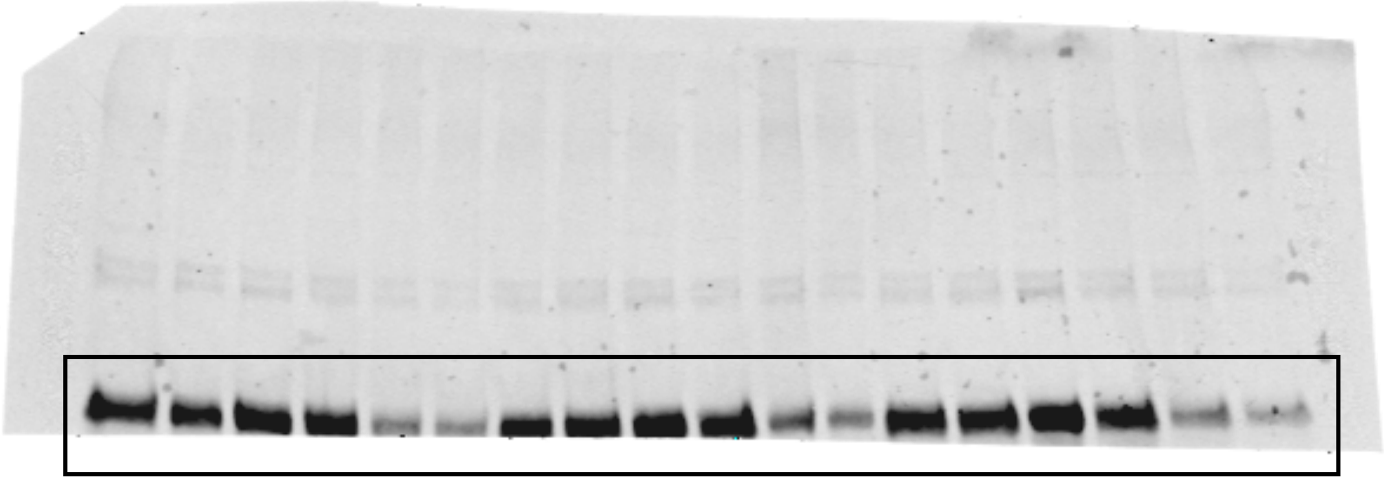

GAPDH

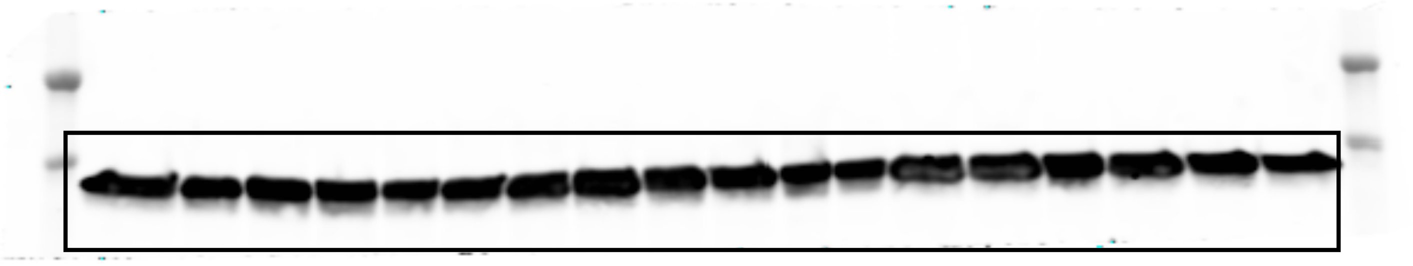

LC3

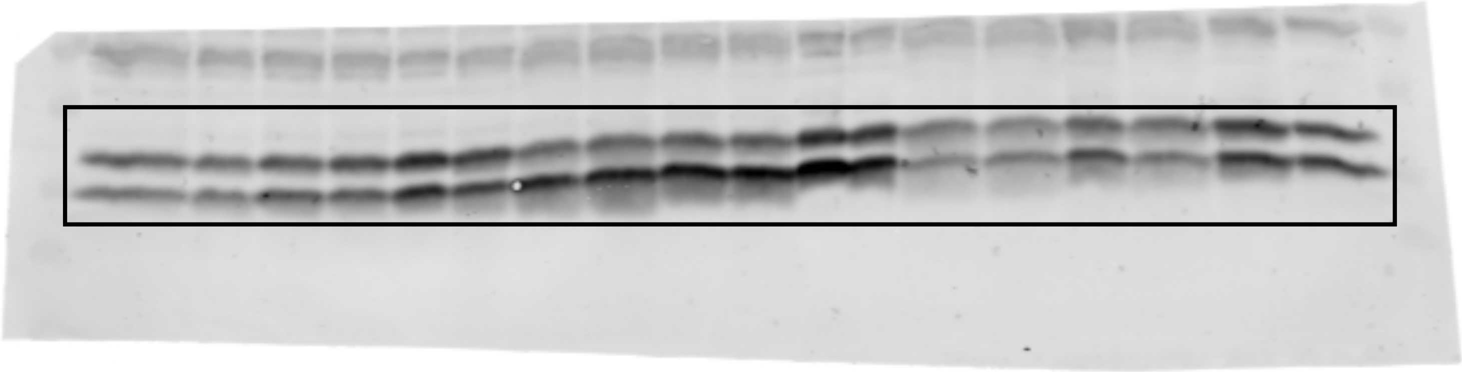

Supplement: Supplementary file 1 — Supplementary Information [file 41467_2021_21931_MOESM1_ESM.pdf]
