# Supplementary material for: Xbp1s-FoxO1 axis governs lipid accumulation and contractile performance in heart failure with preserved ejection fraction
Source: Nat Commun. 2021 Mar 16;12:1684. doi: 10.1038/s41467-021-21931-9 (PMC7966396; doi:10.1038/s41467-021-21931-9)
Supplement: Supplementary file 3 — Reporting Summary [file 41467_2021_21931_MOESM3_ESM.pdf]

nature research

Corresponding author(s): Joseph A. Hill MD PhD

Last updated by author(s): Jan 10, 2021

# Reporting Summary

Nature Research wishes to improve the reproducibility of the work that we publish. This form provides structure for consistency and transparency in reporting. For further information on Nature Research policies, see our Editorial Policies and the Editorial Policy Checklist.

## Statistics

For all statistical analyses, confirm that the following items are present in the figure legend, table legend, main text, or Methods section.

| n/a | Confirmed | |
|---|---|---|
| ☐ | ☒ | The exact sample size (*n*) for each experimental group/condition, given as a discrete number and unit of measurement |
| ☐ | ☒ | A statement on whether measurements were taken from distinct samples or whether the same sample was measured repeatedly |
| ☐ | ☒ | The statistical test(s) used AND whether they are one- or two-sided<br>*Only common tests should be described solely by name; describe more complex techniques in the Methods section.* |
| ☒ | ☐ | A description of all covariates tested |
| ☐ | ☒ | A description of any assumptions or corrections, such as tests of normality and adjustment for multiple comparisons |
| ☐ | ☒ | A full description of the statistical parameters including central tendency (e.g. means) or other basic estimates (e.g. regression coefficient) AND variation (e.g. standard deviation) or associated estimates of uncertainty (e.g. confidence intervals) |
| ☒ | ☐ | For null hypothesis testing, the test statistic (e.g. *F*, *t*, *r*) with confidence intervals, effect sizes, degrees of freedom and *P* value noted<br>*Give P values as exact values whenever suitable.* |
| ☒ | ☐ | For Bayesian analysis, information on the choice of priors and Markov chain Monte Carlo settings |
| ☒ | ☐ | For hierarchical and complex designs, identification of the appropriate level for tests and full reporting of outcomes |
| ☒ | ☐ | Estimates of effect sizes (e.g. Cohen's *d*, Pearson's *r*), indicating how they were calculated |

*Our web collection on statistics for biologists contains articles on many of the points above.*

## Software and code

Policy information about availability of computer code

| | |
|---|---|
| Data collection | VisualSonics Vevo 2100 system - LI-COR Odyssey 3.0 software - BioRad CFX 3 - Zen Software (Zeiss) - Leica Application Suite (LAS) - CODA® High Throughput System - NeoFox, Ocean Optic |
| Data analysis | GraphPad Prism software 8.0 - Simple Western program (ProteinSimple) -LI-COR Odyssey 3.0 software - CFX Maestro Software - Abobe Illustrator CS3 |

For manuscripts utilizing custom algorithms or software that are central to the research but not yet described in published literature, software must be made available to editors and reviewers. We strongly encourage code deposition in a community repository (e.g. GitHub). See the Nature Research guidelines for submitting code & software for further information.

## Data

Policy information about availability of data

All manuscripts must include a data availability statement. This statement should provide the following information, where applicable:

- Accession codes, unique identifiers, or web links for publicly available datasets
- A list of figures that have associated raw data
- A description of any restrictions on data availability

The authors declare that the data supporting the findings of this study are available within the paper and its Supplementary information files. Source data are provided with this paper. Any remaining data that support the results of the study will be available from the corresponding author upon reasonable request.

# Field-specific reporting

Please select the one below that is the best fit for your research. If you are not sure, read the appropriate sections before making your selection.

- [x] Life sciences
- [ ] Behavioural & social sciences
- [ ] Ecological, evolutionary & environmental sciences

For a reference copy of the document with all sections, see nature.com/documents/nr-reporting-summary-flat.pdf

# Life sciences study design

All studies must disclose on these points even when the disclosure is negative.

| | |
|---|---|
| Sample size | No statistical analysis was used to predetermine sample sizes. Estimates were made based on our previous experience with relevant publications cited in the manuscript such as Schiattarella GG et al. Nature 2019 and Tong D, Schiattarella GG et al. Circulation. 2020. For thr assays both in vitro and in vivo, there are generally at least 3-4 experimental/biological replicates for every assay and condition. Experimental approach, availability and feasibility required to obtain statistically significant results were also taken in consideration. |
| Data exclusions | Outlier data points were identified using GraphPad Prism software 8.0 . No data were excluded from the analyses |
| Replication | Data reported in this manuscript were reproduced with at least 3-4 biologically independent replicates for the in vitro experiments and at least 3-4 independent mice per group. All replications attempts were successful. |
| Randomization | Mice were randomly assigned to each experimental/control group. For in vitro experiments, allocation of the different experimental treatments (e.g. viral infection, drug treatment) occurred randomly. In brief, cells were plated and plates were randomly assigned to experimental or control groups. |
| Blinding | Investigators performing echocardiography and performing the analyses was blinded to the genetic model. Investigators were blinded during tissue collection and processing. Molecular assays were performed by individuals who were blinded to the genetic model(s) but not blinded to allocation and outcome assessment. Similar procedures applied for the in vitro work. |

# Reporting for specific materials, systems and methods

We require information from authors about some types of materials, experimental systems and methods used in many studies. Here, indicate whether each material, system or method listed is relevant to your study. If you are not sure if a list item applies to your research, read the appropriate section before selecting a response.

## Materials & experimental systems

| n/a | Involved in the study | |
|---|---|---|
| [ ] | [x] | Antibodies |
| [ ] | [x] | Eukaryotic cell lines |
| [x] | [ ] | Palaeontology and archaeology |
| [ ] | [x] | Animals and other organisms |
| [x] | [ ] | Human research participants |
| [x] | [ ] | Clinical data |
| [x] | [ ] | Dual use research of concern |

## Methods

| n/a | Involved in the study | |
|---|---|---|
| [x] | [ ] | ChIP-seq |
| [x] | [ ] | Flow cytometry |
| [x] | [ ] | MRI-based neuroimaging |

## Antibodies

| | |
|---|---|
| Antibodies used | FoxO1 (#2880, Cell Signaling); FoxO3 (#2497, Cell Signaling); Xbp1s (poly6195, BioLegend); GFP (AB10145, Sigma-Aldrich); STUB1 (#2080, Cell Signaling); Ubiquitin (#3933, Cell Signaling); LC3 (previously developed as in PMID: 17607355); Lamin A/C (05-714, Sigma-Aldrich); PDK4 (previously developed as in PMID: 29540486), OXPHOS cocktail (ab110413, abcam), GAPDH (10R-G109a, Fitzgerald). |
| Validation | All of antibodies were validated by vendors. All validation statements, citations, antibody/antigen details are found on the companies websites. We based specificity on their provided data sheets. For STUB1 antibody we performed the target validation using specific siRNAs. In particular, three sequences of rat Mission-Sigma siRNA (SASI_Rn02_00203179; SASI_Rn01_00039478; SASI_Rn01_00039479 ) were purchased and tested to evaluate the specificity of STUB1 antibody (#2080, Cell Signaling) for STUB1 bands.<br><br>Cell Signaling Technology validation statement: Western blotting remains one of the most common scientific methods for monitoringprotein expression in cells or tissue. The accuracy of western blot results relies heavily of the quality of the primary antibodyemployed in the immunoblotting. Cell Signaling Technology (CST) provides the highest quality primary and secondary antibodiesavailable for western blotting. CST™antibodies are produced in-house and validated extensively according to a rigorous protocol. |

Sigma validation statement: The success of any immunodetection experiment depends on the quality of the antibodies which areemployed. However, antibody reagents vary significantly and when selecting an antibody for a downstream application it is a goodidea to spend some time ensuring that not only has it been tested in the chosen experimental setup but that it also demonstrates therequired specificity, sensitivity and reproducibility. This valuable data is generated during the antibody manufacturing and validationprocess and can be found on the product datasheet with which the antibody is supplied.

Biolegend validation statement: BioLegend antibodies undergo an extensive series of testing to ensure quality at every step in the manufacturing process, as well as maintaining quality after the sale.

Abcam validation statement: Antibodies are validated in western blot using lysates from cells or tissues that we have identified to express the protein of interest. Once we have determined the right lysates to use, western blots are run and the band size is checked for the expected molecular weight. We will always run several controls in the same western blot experiment, including positive lysate and negative lysate. When possible, we also include knock-out (KO) cell lines as a true negative control for our western blots. We are always increasing the number of KO-validated antibodies we provide. In addition, we run old stock alongside our new stock. If we know the old stock works well, this also acts as a suitable positive control.

Fitzgerald statement: Antibodies are raised against a specific target of interest. The primary antibody recognises and binds to the target epitope. At Fitzgerald Industries we offer a range monoclonal antibodies and polyclonal antibodies in both conjugated and unconjugated forms. Our primary antibodies provide a high level of specificity and can be used to measure changes in biomolecules during processes such as phosphorylation, methylation, or glycosylation. Primary antibodies are often employed in the detection of cancer, diabetes, Parkinson's, Alzheimer's and other diseases

## Eukaryotic cell lines

Policy information about cell lines

| | |
|---|---|
| Cell line source(s) | HEK293 (Clontech; cat# 632271) ; Adeno-X 293 cell line was purchased from Clontech (Adeno-X™ 293 Cell Line, cat# 632271) |
| Authentication | Cell line was authenticated by the vendor as the cell lines used in this investigation are well established and widely used. We did not perform any validation except for visual evaluation of morphology. |
| Mycoplasma contamination | Mycoplasma detection was tested negative |
| Commonly misidentified lines (See ICLAC register) | None |

## Animals and other organisms

Policy information about studies involving animals; ARRIVE guidelines recommended for reporting animal research

| | |
|---|---|
| Laboratory animals | Stated in Materials and Methods under "Experimental animals". C57BL/6N mice were used for wild-type (WT) studies. Tetracycline responsive elements (TRE)-Xbp1s mice were crossed with mice harboring a tetracycline transactivator (tTA) transcription factor driven by α-myosin heavy chain promoter (MHC-tTA) to generate mice with cardiomyocyte-specific inducible over-expression of Xbp1s (Xbp1s TG)9,50. Cardiomyocyte-specific FoxO1 knockout (cKO) animals, mice harboring a floxed FoxO1 allele were crossed with α-MHC-MerCreMer transgenic mice. FoxO1-cKO and corresponding control floxed or α-MHC-MerCreMer mice were maintained on an FVB genetic background. Male adult (10/12-week-old) mice were used in the experiments. Mice were maintained on a 12-hour light/dark cycle from 6 AM to 6 PM, 65-75 °F, 40-50% humidity and had unrestricted access to food (#2916, Teklad for CHOW groups and #D12492, Research Diet Inc. for the HFD groups) and water. N [w]-nitro-l-arginine methyl ester (L-NAME; 0.5 g/L, Sigma Aldrich) was supplied in the drinking water or embedded in the chow (custom made #D16082402, Research Diet Inc). HFpEF model was induced by feeding mice with combination of HFD+L-NAME for 5 to 15 weeks as previously described9,10. Pregnant Sprague-Dawley rat were purchased (Charles River Laboratories) and were used for the sole purpose of harvesting primary cardiomyocytes from 1-2-day old rat pups. |
| Wild animals | The study did not involve wild animals. |
| Field-collected samples | The study did not involve samples collected from the field. |
| Ethics oversight | All experiments involving animals conformed to the Guide for the Care and Use of Laboratory Animals published by the US National Institutes of Health (NIH Publication 8th edition, update 2011) and were approved by the Institutional Animal Care and Use Committee of the University of Texas Southwestern Medical Center. All studies were in compliance with all ethical regulations. |

Note that full information on the approval of the study protocol must also be provided in the manuscript.
